# Adherence and eating experiences differ between participants following a flexitarian diet including red meat or a vegetarian diet including plant-based meat alternatives: findings from a 10-week randomised dietary intervention trial

**DOI:** 10.3389/fnut.2023.1174726

**Published:** 2023-06-14

**Authors:** Nicola A. Gillies, Anna Worthington, Larissa Li, Tamlin S. Conner, Emma N. Bermingham, Scott O. Knowles, David Cameron-Smith, Rina Hannaford, Andrea Braakhuis

**Affiliations:** ^1^Discipline of Nutrition, School of Medical Sciences, Faculty of Medical and Health Sciences, The University of Auckland, Auckland, New Zealand; ^2^Department of Psychology, University of Otago, Dunedin, New Zealand; ^3^Smart Foods and Bioproducts Group, AgResearch Ltd., Palmerston North, New Zealand; ^4^School of Environmental and Life Sciences, College of Engineering, Science and Environment, University of Newcastle, Sydney, NSW, Australia; ^5^Digital Agriculture Group, AgResearch Ltd., Palmerston North, New Zealand

**Keywords:** adherence, dietary intervention, eating behaviours, flexitarian, vegetarian, diet satisfaction, behaviour change, young adults

## Abstract

**Background:**

Flexitarian, vegetarian and exclusively plant-based diets are increasingly popular, particularly amongst young adults. This is the first randomised dietary intervention to investigate the health, wellbeing, and behavioural implications of consuming a basal vegetarian diet that additionally includes low-to-moderate amounts of red meat (flexitarian) compared to one containing plant-based meat alternatives (PBMAs, vegetarian) in young adults (ClinicalTrials.gov NCT04869163). The objective for the current analysis is to measure adherence to the intervention, nutrition behaviours, and participants’ experience with their allocated dietary group.

**Methods:**

Eighty healthy young adults participated in this 10-week dietary intervention as household pairs. Household pairs were randomised to receive either approximately three serves of red meat (average of 390 g cooked weight per individual, flexitarian group) or PBMAs (350–400 g per individual, vegetarian group) per week on top of a basal vegetarian diet. Participants were supported to adopt healthy eating behaviours, and this intervention was developed and implemented using a behaviour change framework. Adherence (eating allocated red meat or PBMA, abstaining from animal-based foods not provided by researchers) was continuously monitored, with total scores calculated at the end of the 10-week intervention period. Eating experiences were measured by the Positive Eating Scale and a purpose-designed exit survey, and a food frequency questionnaire measured dietary intake. Analyses used mixed effects modeling taking household clustering into account.

**Results:**

The total average adherence score was 91.5 (SD = 9.0) out of a possible 100, with participants in the flexitarian group scoring higher (96.1, SD = 4.6, compared to 86.7, SD = 10.0; *p* < 0.001). Those receiving red meat were generally more satisfied with this allocation compared to those receiving the PBMAs, even though a leading motivation for participants joining the study was an opportunity to try plant-based eating (35% expressed that their interest in taking part was related to trying plant-based eating). Participants in both intervention groups had increased vegetable intake (*p* < 0.001), and reported more positive eating experiences (*p* = 0.020) and satisfaction with eating (*p* = 0.021) at the end of the 10-week intervention relative to baseline values.

**Conclusion:**

Methods to encourage engagement with the trial were successful, as participants demonstrated excellent adherence to the intervention. Observed differences in participants’ adherence and experiences between flexitarian and vegetarian groups holds implications for the adoption of healthy, sustainable dietary patterns beyond this study alone.

## 1. Introduction

Young adulthood is a life stage characterised by major social, biological, and environmental changes which can have both positive and detrimental influences on eating and health trajectories (1–3). Flexitarian and exclusive plant-based diets are increasingly popular amongst young adults in particular (4), a transition driven by multiple factors such as improving health, cost-effectiveness, environmental awareness, and ethical awareness (5).

The health, wellbeing, and behavioural implications of including low-to-moderate amounts of red meat typical of flexitarian dietary patterns remains uncertain in young adults. This is a pertinent issue given the increasingly wide spread availability and promotion of plant-based meat alternatives (PBMAs) in the market (6). At the same time, experts are advising caution regarding the role of such products in healthy and sustainable diets given their highly processed nature (7) and the reductionist approach of mimicking complex wholefood sources (6).

A critical aspect of any healthy, sustainable dietary pattern is how well an individual or group accepts and can adhere to those recommendations (8). For example, evidence from weight loss intervention studies suggests that adherence to a diet is more relevant than differences in the macronutrient composition of two different diets for long-term maintenance of weight loss (9, 10). In the context of dietary intervention studies more broadly, understanding how well participants have adhered to the intervention prescribed is necessary for subsequent interpretation of study outcomes. However, measures of adherence are infrequently reported and remain challenging in free-living individuals compared to tightly-controlled feeding studies (11). Increased attention to monitoring and reporting of adherence is needed to improve the quality and validity of dietary intervention trials (11).

Adherence is inherently connected to eating behaviours and experiences, yet there is a paucity of research considering these psychological factors alongside measures of adherence in dietary intervention trials. Notably, Landry et al. demonstrated how this can be achieved by combining qualitative and quantitative measures of study diet satisfaction in their analysis of adherence to a Ketogenic or Mediterranean diet in individuals with pre-diabetes or diabetes (12). Although not strictly relating to dietary adherence, baseline psychosocial factors like food addiction or poorer health-related quality of life have also been associated with study attrition in the DIETFITs weight loss trial (13). While “adverse” behaviours (e.g., symptoms of food addiction) have been associated with failure to complete the intervention or achieve desired outcomes in overweight individuals (13), more positive eating behaviours or attitudes may better complement measures of adherence in a healthy study population. For instance, the positive eating scale focuses on more general eating behaviours without a focus on eating pathology (14), but as a relatively new instrument has not been used alongside measures of adherence in dietary intervention trials to date. There is scope and promise for combining psychosocial factors and measures of adherence in dietary intervention trials to not only build a more in-depth picture of adherence, but to help inform the translation of dietary recommendations that individuals can adopt and maintain.

This is the first analysis of the PREDITION (pRotEin Diet SatisfacTION) trial, a 10-week dietary intervention in young adults measuring physiological and psychological responses to a healthy diet containing moderate amounts of red meat (approximately 3 serves per week, with an average weekly allocation of 390 g cooked red meat) or PBMAs (3 serves per week, with a weekly allocation of 350–400 g of packaged PBMAs) in young adults. In this paper we report on adherence to the allocated dietary intervention, nutrition behaviours and psychosocial factors related to eating experiences.

## 2. Methods

### 2.1. Trial design and setting

This was a parallel-group randomised dietary intervention, comprised of a 2-week lead-in period (t-_2_), a baseline assessment at week-0 (t_0_), 10 weeks of dietary intervention, and a post intervention follow-up at 22 weeks after participants started the intervention (t_22_). The protocol was developed in accordance with the Standard Protocol Items: Recommendations for Interventional Trials (SPIRIT) guidelines and has been fully disclosed in an advance publication (15).

Participants were recruited as a “household unit,” which refers to a pair of individuals (spouses/partners or flat mates/housemates) who cohabit. Participants were recruited in pairs as a means to encourage adherence and completion of the study. The 40 pairs were organized into 8 subgroups, each including 5 household units. Timelines for each subgroup were pre-defined (i.e., intervention start dates) and subgroups were formed chronologically, with participants allocated to join the next available subgroup starting following enrollment into the study. The only reason participants would not join the next available subgroup is in the case of schedule clashes (e.g., if they are travelling at the scheduled start date), in which cases they would join the first available subgroup their schedule permitted.

Subgroups (*n* = 10 participants per subgroup) were assigned to the red meat or PBMA allocation using a random allocation sequence (1:1 ratio) generated through https://www.randomizer.org. Random allocation was performed at the level of subgroups rather than household pairs due to the intervention design, requiring subgroups of participants on the same intervention arm to receive and engage with the online nutrition support package at the same time. Researchers responsible for participant recruitment and assessment were blinded to allocation throughout the period of subgroup formation and until participants started their 2-week lead in period, at which point arrangements for food delivery were required. Due to the format of the foods, participants were not blinded to their intervention although they were not made aware of this until after baseline assessment.

Recognizing that there are many interpretations of the terms vegetarian and flexitarian, in this study we defined vegetarian as ovo-lacto vegetarian and flexitarian as ‘a vegetarian diet with moderate amounts of red meat’ (16). Although there is no universal definition for the term flexitarian, a flexitarian is generally agreed to refer to as a semi-vegetarian or a ‘meat reducer’ (17). This can include individuals who regularly consume smaller quantities of meat compared to a standard Western Diet, through to those who only eat meat on occasion (16). The place of red meat in a flexitarian diet is poorly defined in the literature, and we acknowledge that our use of the term flexitarian in this study aligns with some definitions [e.g., “one that is primarily vegetarian with the occasional inclusion of meat or fish,” without specifying whether meat refers to red or white meat sources (17)], but not others which focus on red and processed meat reduction (8).

Primary and secondary outcomes were measured at week-10 (t_10_, primary endpoint). Further exploratory analyses were conducted for surveys completed at t_22_. The primary outcome of the PREDITION trial is change in concentrations of polyunsaturated fatty acids in erythrocyte membranes post-intervention, while the current manuscript includes secondary outcomes of adherence, dietary intake, and subjective experience to the diet. Any potential effects of a flexitarian or vegetarian diet are only as worthwhile as the participants adherence to the intervention. Findings are reported in accordance with the Consolidated Standards of Reporting Trials (CONSORT) 2010 guidelines (18).The trial is registered with ClinicalTrials.gov NCT04869163; https://clinicaltrials.gov/ct2/show/NCT04869163, approved by the New Zealand Ministry of Health’s Health and Disability Ethics Committees (20/STH/157), and conducted in accordance with the principles of the Declaration of Helsinki and relevant institutional regulations.

The trial was conducted from the University of Auckland Clinical Research Centre in Auckland, New Zealand. Participants were required to attend 4 research visits in total – one screening visit prior to enrollment, and three visits during the study at t_0_, week-5 (t_5_) and week-10 (t_10_). Due to COVID-19 disruptions, we were unable to complete t_5_ assessments for all participants and this timepoint was omitted from further analyses. In-person testing occurred between May 2021 and May 2022, with remote surveys for the week-22 follow-up completed by August 2022. Due to the ongoing nature of COVID-19 disruptions, participants entered their 10-week interventions in a staggered overlapping series. Retrospectively, this has been defined as four ‘cohorts’ each comprising 2 sub-groups (*n* = 20 participants) balanced for intervention allocation. Cohort 1 entered the study in May–June 2021, Cohort 2 in August 2021, Cohort 3 in February 2022 and Cohort 4 in March 2022 (Supplementary File 1).

Periods of the PREDITION trial ran while COVID-19 was not present in the community (Cohort 1), during strict nationwide lockdowns (Cohort 2), and while COVID-19 was present in the community but people who were not ill could be more free-moving (Cohorts 3 and 4). To monitor the effects of the pandemic on study outcomes, researchers recorded if and when participants were having to isolate at home. This enforced isolation captures both a positive diagnosis of COVID-19 for the participant and situations where a household member has the positive diagnosis. Isolation during the study period was treated as a binary variable (yes/no). A summary of the cohort timeline and relevant COVID-19 restrictions is provided in the Supplementary File 1.

### 2.2. Participants

Participants were aged 18–35 years who in the last 2 months consumed at least 2–3 meals per week containing meat of any description (red or white fleshed meat, fish or seafood), were willing to consume both red meat and PBMAs for the purposes of the trial, owned a mobile phone with a camera and were proficient with using Facebook and Facebook messenger. Potential participants who use dietary supplements were required to abstain for the month prior to the study beginning, and women confirmed that they were not pregnant, nor intending to become pregnant during the trial.

Individuals with chronic health conditions, obesity (BMI ≥ 30 kg/m^2^), hyperlipidemia, history of anosmia and ageusia (issues with smell and taste), use of recreational drugs or medications (except for contraception or occasional NSAIDs and antihistamine use), or who smoke tobacco were excluded from participating. Given the routine monitoring of food intake and subjective experience to food required in this study, individuals’ eating behaviours were screened with the revised three-factor eating questionnaire (TFEQ-R18) (19). The TFEQ more broadly describes eating behaviours, rather than explicitly screening for disordered eating. After TFEQ scores had been transformed to a 0–1 scale, those with a score ≥ 0.75 were excluded from participation. Although there is no widely used cut-off score for the TFEQ, this was deemed relevant for the purpose of the study by the research team (including psychologists and dietitians), but is not considered diagnostic in any sense.

Recruitment occurred via advertisements with posters placed around the University of Auckland and using social media websites. Potential participants completed a web-based eligibility screening, and participants were then invited to meet with the research team in person where eligibility was confirmed. Written informed consent was obtained from all participants after they had received a complete description of the study, and opportunity for discussion with researchers. Participants were introduced to the Easy Diet Diary software at this screening visit, further described in section 2.4.1 below.

### 2.3. Dietary intervention

Household units received regular deliveries of either red meat (herein referred to as “flexitarian group”) or PBMAs (herein referred to as “vegetarian group”) each fortnight. These inputs were well-regulated additions to the basal vegetarian diet participants were required to maintain throughout the 10-week intervention. Both groups could consume eggs and dairy products, but not chicken, pork or fish, and no red meat other than that supplied by the researchers to the flexitarian group.

The average quantity of uncooked red meat provided was 540 g per person per week (approximately 390 g cooked weight), with a range of 380–650 g. This quantity conforms to the latest international recommendations for maximum intake from the World Cancer Research Fund and the Eating and Activity Guidelines in New Zealand of 500 g cooked red meat per week, or approximately 700–750 g uncooked red meat (20). The meat was pasture-raised beef and lamb butchered and packaged to specifications in New Zealand, and cuts included beef mince, beef steak, lamb rack and lamb leg which explains the variability in the quantity of red meat provided each week.

The PBMAs were locally available soy- and pea-protein based commercial products which were selected based on similarity of quantity (3 serves, 350–400 g cooked weight), form (“beef style” mince and patties), and macronutrient composition (with a focus on total protein and fat) (15). For practicality, we describe this as “approximately 3 serves” of red meat or PBMA provided each week, but it is acknowledged that participants may prepare and consume these foods according to their preference for serving size.

In addition to their allocated red meat or PBMA, household units received a weekly vegetarian meal kit delivery (Woop Ltd., New Zealand) containing complete ingredients and recipe cards for three evening meals. Participants had a degree of flexibility within their diet for other meals, but were provided with a cookbook designed for the PREDITION trial by research dietitians and online nutrition videos/support to facilitate healthy meal choices and preparation. This nutrition support was developed and implemented by registered dietitians using an existing behaviour change framework (21). The goal of this nutrition support was to encourage participants to consume a healthy basal vegetarian diet. According to the latest New Zealand Adult Nutrition Survey, undesirable eating behaviours of young New Zealand adults included inadequate intake of fruits, vegetables, and wholegrains, excess consumption of sugar-sweetened beverages, and high consumption of food purchased outside of the home (21, 22). Irregular meal patterns and high rates of meal skipping are another common feature of poor dietary habits in young adults (23). Target behaviours for the nutrition support package in the PREDITION trial therefore included meeting local recommendations for fruit (2 serves/day) and vegetables (5 serves/day), choosing wholegrains options (at least once/day), minimizing intake of discretionary foods (e.g., fast food, fizzy drinks), and establishing a regular meal pattern. The nutrition support package was more generalized advice to support the adoption of healthy dietary behaviours, and participants did not receive a diet plan or recommendations around individual energy intake.

### 2.4. Assessments

#### 2.4.1. Adherence to the dietary intervention

Participants agreed to record their dietary intake for the course of the 10-week intervention in the Easy Diet Diary app (Xyris Software (Australia) Pty Ltd). Participants downloaded the Easy Diet Diary app onto their personal mobile phone, which was connected to research dietitians through an invitation link sent at t-_2_. This app enables researchers to see all data entered in real time, allowing adherence to be routinely monitored and prompted during the intervention. Instructional support on using the app was provided at t-_2_ and reinforced at t_0_. From t_0_, participants were required to record all meals, snacks, and beverages into the app as a daily record either as text (2 days/week, on Sunday and Monday) or photos (5 days/week, on Tuesday – Saturday). Participants received regular, standardised text reminders to complete their diaries three times a week. The implementation method was mixed to allow for complete adherence monitoring while minimizing participant burden (ease of photo entry), and still allowing for sufficient data collection for future exploratory analyses from the Easy Diet Diary app (full text recording 2 days/week).

Adherence was assessed according to information logged in the Easy Diet Diary app as a daily record (either as photos or text entry) against two criteria, whether participants (A) Consumed all red meat or PBMAs provided each week, and (B) Abstained from red or white meats and seafood (other than that provided to the flexitarian group by the researchers). Adherence was monitored twice a week by researchers. If participants had not provided sufficient evidence of consuming allocated intervention foods, or appeared to eat animal-source foods other than that provided by the research team within a 3–4 day period the individual participant was sent a text message for clarification. Both the daily record and this additional information sent to researchers was used to quantify adherence (i.e., a participant might confirm that they had only consumed 2 serves of PBMA rather than the allocated 3 serves). If a participant was non-adherent for three consecutive periods, the household unit were contacted and would be discontinued from the study if adherence did not improve in the next reporting period. This regular review/contact by researchers encourages but does not force adherence in free-living individuals. Our goal in extensively monitoring adherence was to ensure that participants were adequately compliant for subsequent physiological and psychological analyses to be valid, with accurate information to substantiate this. The cut-off for exclusion is still relatively loose, providing some degree of freedom as to how strictly participants adhered to adherence guidelines – as is necessary for free-living individuals. This allows for a spectrum of adherence throughout the study, from those who are perfectly adherent throughout the entire 10-week intervention, to those who may be considered non-adherent for just one reporting period, through to those who may cycle between non-adherence and adherence from week to week.

A total adherence score was calculated at the end of the intervention from the two simple criteria described above. Each week of the intervention participants received a score of 1 if they consumed all of their allocated red meat or PBMA, and a further score of 1 if they abstained from consuming other meat products, or a score of 0 if they did not. The maximum adherence score was 20 points across the 10-week intervention. This was converted to a scale from 0 to 100 for subsequent analyses, with a higher score reflecting better adherence. The proportion of participants meeting adherence requirements each week (both consuming all allocated red meat or PBMA, and abstaining from consuming other meat products) was also monitored over time.

#### 2.4.2. Dietary intake

Participants completed a self-administered short-form version of the Otago Food Frequency Questionnaire (FFQ) prior to the intervention commencing (t-_2_), at t_10_ (reflecting intake during the intervention), and at the week-22 follow-up. This 57-item semi-quantitative FFQ is validated in New Zealand to assess overall nutrient intake over a 3-month period (24). Additional free-text entries were included to assess how many serves of core food groups were consumed each day (fruits, vegetables, cereals) or week (meat and poultry, seafood), with standard serving size examples provided.

#### 2.4.3. Positive eating scale

The Positive Eating Scale (PES) consists of 8 items on a 4-point Likert score (14). This questionnaire captures participants’ experiences of eating, and participants were asked to rate their eating experiences “over the past week” for this study. There were 4 items measuring satisfaction (e.g., I eat in a way that makes me feel good.) and 4 items measuring pleasure (e.g., Eating is a pleasure for me.). Mean scores were calculated for the total positive eating score (all 8 items), satisfaction with eating sub score (4 items), and pleasure when eating sub score (4 items). Scores range from 1 to 4, with higher scores indicating a favourable response.

The PES questionnaire was completed prior to the intervention commencing (−t_2_, t_0_), at weeks 2, 5, 7, and 10 of the intervention, and at the week-22 follow-up. For statistical analysis of PES scores, we aimed to use the average of –t_2_ and t_0_ questionnaires as the baseline measurement. However, for participants in cohorts 3 and 4 (*n* = 38), the t_0_ questionnaire was omitted from baseline scores as it was completed after allocation was revealed. This was a disruption from COVID-19 which required the research team to minimize the time spent in person during clinic visits. To ensure that all baseline measurements are pre-allocation, we have adopted this conservative approach.

#### 2.4.4. Baseline health, demographic, and anthropometric analysis

##### 2.4.4.1. Three factor eating questionnaire

The Three-Factor Eating Questionnaire (TFEQ) evaluates three cognitive and behavioural domains of eating – restrained eating, uncontrolled eating, and emotional eating. A self-administered short-form (TFEQ-18) was completed online during participant screening to exclude participants with potentially disordered eating behaviours (TFEQ ≥0.75 after raw scores transformed to a 0–1 scale). The TFEQ scores of enrolled participants were subsequently considered as a covariate in statistical analyses, with scores ranging between 0 and 0.74 and higher scores indicating greater cognitive restraint (control over food intake to influence body weight/shape), disinhibition (episodes of loss of control over eating), and emotional eating (25).

##### 2.4.4.2. Self-efficacy questionnaire

Participants completed a self-efficacy questionnaire during screening, which was purpose-designed for the PREDITION trial. The questionnaire consists of 8 items on a 5-point Likert score, reflecting how confident participants are in achieving behaviours necessary for this dietary intervention [e.g., “How confident are you in eating your allocated intervention food (red meat/plant-based meat alternatives?,” or “How confident are you in cooking meals from a delivery kit?”)]. Questions pertained to adherence to healthy eating behaviours, adherence to the flexitarian or vegetarian dietary patterns, and cooking skills. Scores ranged from 1 to 40, with higher scores indicating great self-efficacy for successfully completing the PREDITION trial.

##### 2.4.4.3. Anthropometry

Height and weight were measured at participants’ t_0_ clinic visit. Height was measured using a free-standing stadiometer, with the average of two measurements recorded. Weight was measured using an A&D Scale (HW-PW-200-FG, A&D Medical, Australia). Body mass index (BMI) was calculated as weight (kg)/height (m)^2^.

##### 2.4.4.4. Health and demographics questionnaire

Information on participants’ health and demographic characteristics was collected during screening. Items on the questionnaire relevant to the current analysis include age, sex, highest level of education achieved, frequency and intensity of exercise, and self-rated health (excellent, very good, good, or fair/poor).

#### 2.4.5. Exit survey

Participants completed the exit survey following their final assessments at week 10. Quantitative analysis included questions around satisfaction with the meal delivery kits provided, satisfaction and enjoyment of the PBMAs or red meat provided, and ease of adhering to a predominantly vegetarian diet. Responses were on a 5-point scale, from “strongly disagree” through to “strongly agree.”

Open-ended text boxes were also included in this survey for qualitative analysis. The following questions were asked; “Can you please comment on what you did/did not enjoy about eating your plant-based meat alternatives or red meat during the study,” “what did you like best about participating in this study,” and “what did you like least about participating in this study.” Participants were also asked what their “main interest in taking part in the study” was, and were able to record multiple interests in the study.

### 2.5. Statistical analysis

Sample size estimates for the PREDITION trial were calculated for the primary outcome of change in concentrations of polyunsaturated fatty acids in erythrocyte membranes post-intervention, leading to a sample size of 63 required to detect a small effect (Cohen effect size of 0.2) (15). A sample size of 80 was recruited to allow for drop-outs. Power calculations were not performed for the secondary outcomes reported here, as they are largely descriptive.

All available data from participants who completed the 10-week dietary intervention were included in analyses on the primary endpoint (*n* = 78). The amount of missing data was small, with a 99.0% complete data set (464 of the 468 questionnaires were completed). Supplementary Table 1 provides further detail of response attrition across the 10-week intervention. Fewer participants responded to the week-22 follow-up survey (82%), with a 96.7% complete data set in total. Data were analyzed using statistical packages of R software (R Core Team, version 4.1.1, 2021).

Logistic regression was used to assess whether there was evidence that the probability a participant adhered to the intervention was affected by which intervention group they were in, or any of their measured demographic (age, sex, education, cohabiting with partner or flat mate), health (level of exercise, self-rated health, isolating or not due to COVID, TFEQ score), and anthropometric characteristics (BMI). The statistical significance of all possible combinations of these covariates were assessed using the corrected Akaike’s information criterion (AICc), with the model with the lowest AICc value chosen (Supplementary Table 3). The model was fitted as a mixed effects model, with the covariates chosen by the AICc as fixed effects, and household unit nested within cohort as random effects. The normality assumption regarding conditional distribution of errors was checked and satisfied through monitoring residual plots and histograms of the residuals (Supplementary Figure 2).

Linear mixed effect models were used to assess change in PES scores. As above, AICc was used to assess the statistical significance of all possible combinations of variables for predicting each of the measured PES scores (Supplementary Table 4) and the final model was fit with household pair nested within cohort as random effects. The normality assumption regarding conditional distribution of errors was again monitored for the changes to PES scores (Supplementary Figure 2).

Linear mixed effect models were used to assess changes in dietary intake, with time and intervention group fit as fixed factors, including an interaction term between time and intervention group, and subject set as a random factor. For significant interaction terms, the Tukey adjustment was applied to correct for multiple comparisons. Normality of outcome variables was assessed graphically through histogram plots, with all achieving an approximately normal distribution. A more simple analysis approach was taken for this dietary analysis compared to other behavioural outcomes, as the purpose is to understand absolute changes to dietary intake that occurred during the intervention rather than understanding the effect of covariates on these changes.

The analyses for PES scores and dietary intake were repeated with the sub-set of participants who completed the week-22 follow-up survey (*n* = 64) to evaluate whether any changes to dietary intake were maintained after the intervention was complete.

The exit survey included open-ended responses which were transcribed and entered in nVivo [release 1.5.2 (946)] for inductive qualitative analysis (26). Responses to each question were sorted into codes, which were then categorized into overarching themes. A second researcher confirmed the data each theme contained. Additionally, for the question relating to interest in the study each finalized theme was attributed a number and the raw interest data was coded with the number of the themes it contained.

## 3. Results

### 3.1. Participant flow and characteristics

**Figure 1:**
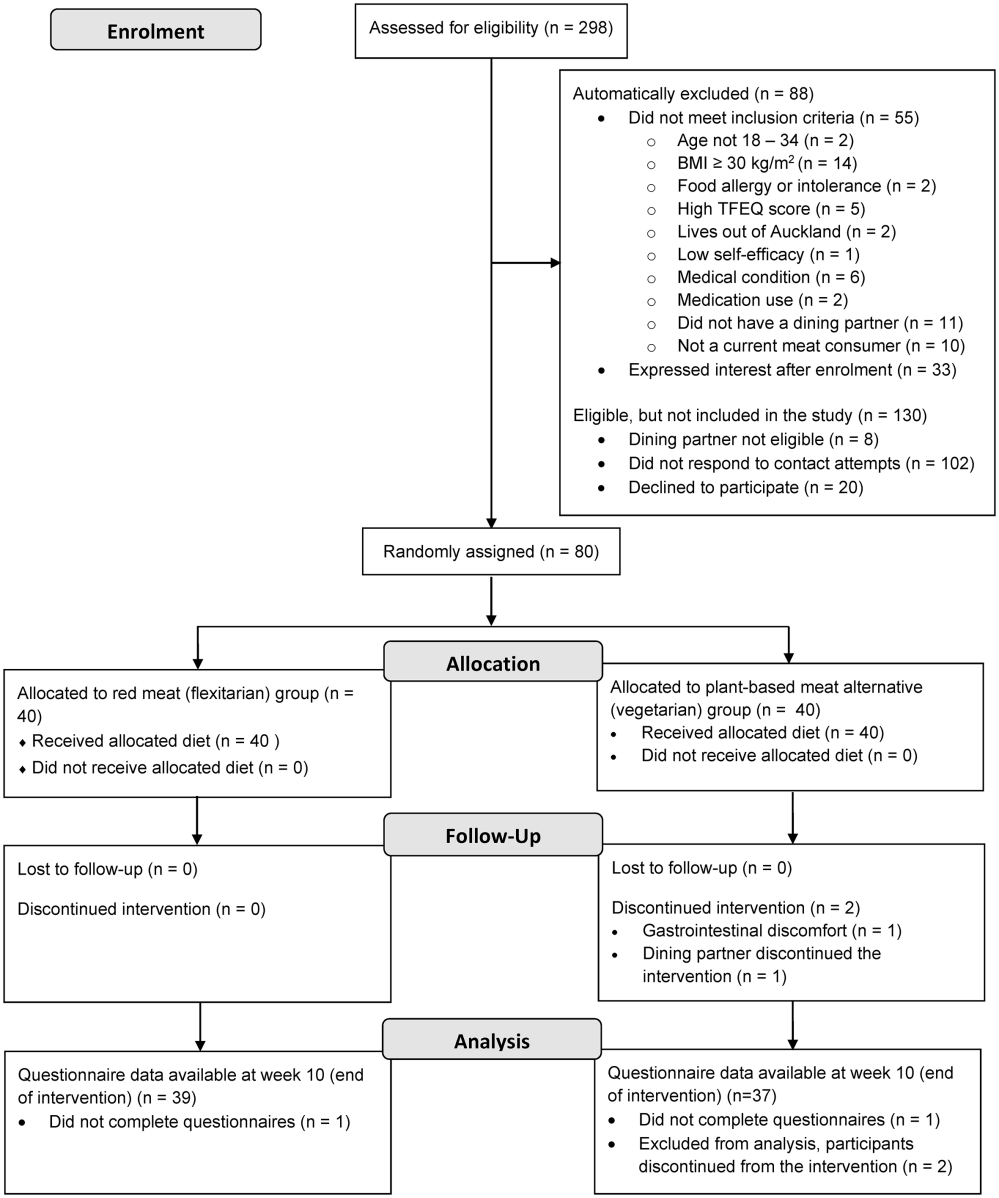
Consolidated standards of reporting trials (CONSORT) flow diagram of participant enrolment and random assignment to and analysis of study intervention groups for the current analysis of the PREDITION trial. BMI, Body mass index; TFEQ, Three factor eating questionnaire.

Of 298 potential participants, 80 were enrolled as household pairs and formed 8 subgroups each containing 5 household pairs (*n* = 10). Subgroups were randomly assigned to either the flexitarian (*n* = 40) or vegetarian (*n* = 40) allocation groups. A total of 78 participants completed the 10-week intervention (flexitarian, *n* = 40; vegetarian, *n* = 38). As shown in Figure 1, two participants were withdrawn at week 5 of the intervention.

Baseline characteristics of participants who completed the 10-week intervention are presented in Table 1. Briefly, participants were 55% female with an average age of 25.8 ± 4.3 years. Participants were well educated, with 72% having achieved university-level education, and most rated their health as very good. Differences in baseline characteristics between intervention groups should be noted, with Table 1 showing that the flexitarian group had more females, a greater proportion of participants who had achieved postgraduate education and a greater proportion of participants who self-reported as having excellent health.

**Table 1:** Participant characteristics.

		Total population (*n* = 78)	Flexitarian group (*n* = 40)	Vegetarian group (*n* = 38)
Age		25.8 ± 4.3	25.9 ± 3.6	25.7 ± 4.9
Sex				
	Females	43 (55%)	25 (63%)	18 (47%)
	Males	35 (45%)	15 (38%)	20 (53%)
Education				
	University – postgraduate	23 (30%)	15 (38%)	8 (21%)
	University – undergraduate	33 (42%)	17 (43%)	16 (42%)
	Secondary school or below	22 (28%)	8 (20%)	14 (37%)
Relationship of household unit^1^				
	Housemates	30 (39%)	16 (40%)	14 (37%)
	Partners	48 (61%)	24 (60%)	24 (63%)
BMI		23.9 ± 3.0	23.5 ± 2.7	24.4 ± 3.2
Level of exercise^2^				
	Vigorous	29 (37%)	16 (40%)	13 (34%)
	Moderate	42 (54%)	22 (55%)	20 (53%)
	Sedentary/none	7 (9%)	2 (5%)	5 (13%)
Self-reported health				
	Excellent	15 (19%)	10 (26%)	5 (13%)
	Very good	32 (41%)	18 (45%)	14 (37%)
	Good	31 (40%)	12 (31%)	19 (50%)
	Fair/poor	0 (0%)	0 (0%)	0 (0%)
Self-efficacy		37.1 ± 3.6	36.6 ± 4.3	37.7 ± 2.7
TFEQ		0.52 ± 0.09	0.51 ± 0.08	0.52 ± 0.10

Data is presented as mean ± standard deviations for continuous variables or n (%) for categorical variables. BMI, Body Mass Index; TFEQ, Three factor eating questionnaire.

1 Partners refers to participants in a relationship, housemate refers to participants who cohabit but are not in a relationship.

2 Level of exercise refers to self-reported intensity of exercise that participants regularly engage in.

### 3.2. Participant adherence

Adherence to the dietary intervention (eating the allocated meat or PBMA and abstaining from other meat products) was excellent. Mean adherence scores in the total population were 91.5 (SD = 9.0) out of a possible 100, and individual participant scores ranged between 65 and 100.

**Figure 2:**
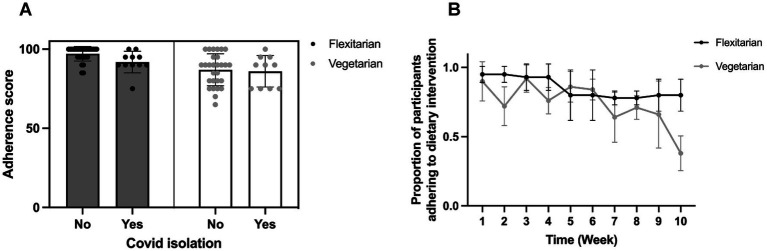
Summary of adherence to the dietary intervention. **(A)** Total adherence scores according to intervention group and whether participants were isolating for COVID-19 requirements, and **(B)** Changes over time in the proportion of participants meeting adherence requirements each week.

Logistic regression models were used to evaluate differences in adherence scores between intervention groups, taking COVID-19 isolation and TFEQ scores into account as covariates (Supplementary Table 3 provides detail on the model selection process). Participants in the vegetarian group had 3.6 times lower odds (*p* < 0.001) of adhering to the dietary intervention (Figure 2), with average adherence scores of 86.7 (SD = 10.0) compared to 96.1 (SD = 4.6) in the flexitarian group (Table 2). There was also a marked decline in the proportion of participants meeting adherence requirements from week 7 onwards in the vegetarian group, as shown in Figure 2. COVID-19 isolation had a significant effect on adherence, such that participants who were isolating were 2.6 times less likely to meet adherence requirements than those who did not have to isolate during the study (*p* = 0.021, Table 2).

**Table 2:** Estimates derived from logistic mixed effect models comparing differences in adherence scores between intervention groups.

	Odds ratio	Estimate (SE)	*t*	*df*	*p* value
Vegetarian group^1^	0.28	−1.28 (0.31)	−4.11	29.31	**<0.001**
COVID-19 isolation^2^	0.38	−0.98 (0.39)	−2.51	40.26	**0.016**
TFEQ score	5.11	1.63 (1.19)	1.38	55.64	0.174

Significant findings (*p* < 0.05) are highlighted in bold text.

TFEQ, Three factor eating questionnaire.

1 Odds of vegetarian group relative to flexitarian group.

2 Odds of having to isolate because of COVID-19 relative to participants who did not.

### 3.3. Positive eating experiences

Positive eating experiences increased from baseline to the end of the 10-week intervention in both intervention groups for both the PES total score (baseline: 3.14, SD = 0.42; t_10_: 3.25, SD = 0.49, time effect *p* = 0.020) and the satisfaction subscale (baseline: 3.00, SD = 0.44; t_10_: 3.18, SD = 0.47, time effect *p* = 0.021; Supplementary Figure 1). A trend towards greater pleasure subscale scores in the flexitarian group was found at week-10 (interaction effect, *p*-value 0.084). Differences in total PES scores were seen between intervention groups at baseline, with the flexitarian group maintaining higher scores across all time points (intervention effect, *p* = 0.021). These effects were found while accounting for covariates such as exercise, TFEQ scores, self-efficacy scores, and self-reported health as detailed in Supplementary Table 2.

A similar pattern was seen in the sub-group of participants who completed week-22 surveys (Supplementary Table 5). Both total (time effect, *p* = 0.025) and pleasure subscale (time effect, *p* = 0.024) PES scores had increased by week-10 but scores had returned to baseline levels by t_22_. Both total (intervention effect, *p* = 0.023) and pleasure subscale (intervention effect, *p* = 0.033) PES scores were higher in the flexitarian group at baseline, which was maintained across all time points.

### 3.4. Participant experiences during the intervention

**Figure 3:**
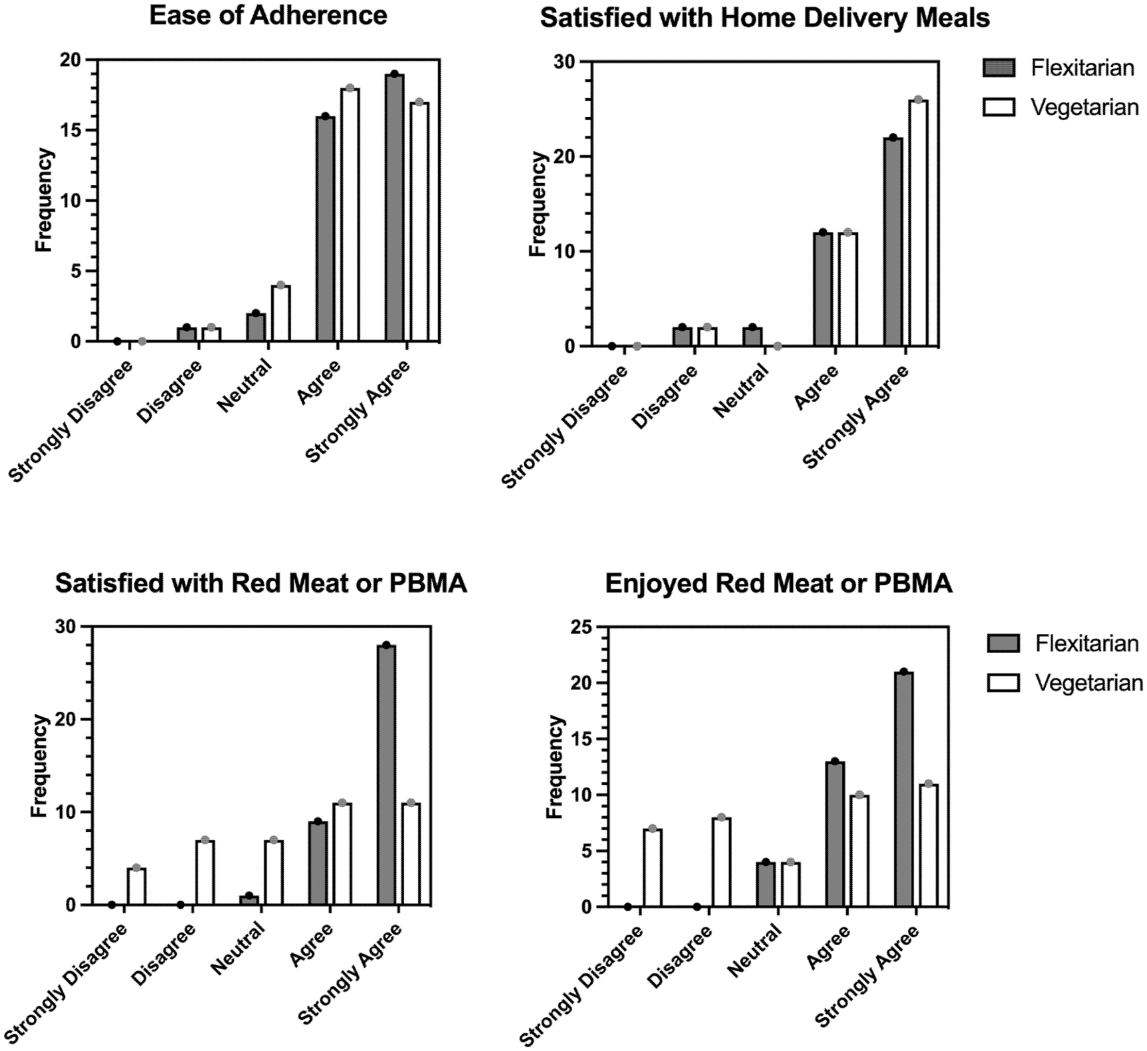
Responses on the Exit survey according to intervention group. The questions participants responded to were: “It was easy for me to adhere to a predominantly vegetarian diet with some plant-based meat alternatives/red meat, “I was satisfied with the Woop meal kits provided to me during the study,” “I was satisfied with the plant-based meat alternatives/red meat provided to me during the study,” “I enjoyed eating the plant-based meat alternatives/red meat provided during this study.” Participants responded on a 5-point Likert scale from strongly disagree to strongly agree, with the frequency of participants reporting each response category presented. PBMA, plant-based meat alternative.

The study population was satisfied with the Woop meal kit deliveries, with 92% agreeing or strongly agreeing with this statement. Similarly, most found it easy to adhere to a predominantly vegetarian diet, with 91% agreeing or strongly agreeing to this statement. Responses to both questions were very similar in the vegetarian and flexitarian groups, but there was a greater split in their satisfaction and enjoyment of their allocated red meat or PBMAs. The flexitarian group was largely satisfied with the red meat and enjoyed eating it (90% agreeing or strongly agreeing), whereas the vegetarian group had a broader range of responses with 58% either agreeing or strongly agreeing with both enjoyment and satisfaction statements (Figure 3).

Qualitative analysis of responses to what participants did and did not enjoy about their allocated red meat or PBMA identified five key themes: enjoyed red meat quality, disliked red meat quantity, like and dislike of taste of PBMAs, and disliked lack of variety of allocated red meat or PBMA, with responses summarized in Table 3.

**Table 3:** Summary of qualitative responses regarding participants’ enjoyment of their intervention allocation.

Theme^1^	Description	Example
Enjoyed red meat quality	Participants in the flexitarian group enjoyed the quality of the red meat provided.	Really good quality red meat provided - having different cuts each week was good too. Amazing quality which meant we really looked forward to it
Too much red meat	Some participants in the flexitarian group did not enjoy having to consume the quantity of red meat provided.	There was a lot of volume, which often made it hard to me to fit all the red meat in. Given we do not eat much meat usually, it was quite tasty at the start, but by the end it felt like a lot of meat
Lack of variety	Participants started to feel dissatisfied with the lack of variety of meat and PBMAs in both the flexitarian and vegetarian groups, respectively.	I did not enjoy eating only one type of meat (red meat) for the whole study I got sick of it towards the end (mostly the steaks) but it was good having the variety, e.g., Minced and lamb. The sameness of the meat alternatives was off putting. By week 3–4 I was over having to try and be creative with the meat alternatives
Disliked taste of plant-based alternatives	Some participants in the vegetarian group did not enjoy the taste of the plant-based meat alternatives.	Did not enjoy plant-based meats, in particular the flavour which was very hard to disguise. The plant-based meat alternatives did not taste that great
Like taste of plant-based alternatives	Some participants in the vegetarian group enjoyed the taste of the plant-based meat alternatives.	I thought the plant meat was surprisingly tasty I really enjoyed eating the plant-based burger patties

1 Data was entered in nVivo for inductive qualitative analysis.

Open-ended text responses provided insight into participants’ interest in the study. Qualitative analysis identified four themes that covered 81% of the data: opportunity to eat healthily (*n* = 23); opportunity to try plant-based eating (*n* = 27); free food (*n* = 31); and health checks (*n* = 23). Other reasons that were not included in these main codes were receiving healthy eating advice, partner signed up, and research-related reasons. Of note, interest in trying a plant-based diet was a leading motivation for participants volunteering to take part in the PREDITION trial, stating that they would like to “try (a) vegetarian diet” and had an “interest in plant-based eating” and “wanted to eat less meat.”

### 3.5. Changes to dietary intake during the intervention

Vegetable intake increased in the total population from t_0_ to the end of the 10-week dietary intervention (*p* < 0.001), moving closer towards government recommendations of 5 (female) – 6 (male) serves/day (27) (Table 4). Participants in the flexitarian group consumed an average of 3.3 (SD = 0.5) serves/day of red meat during the study (allocation provided by researchers), which was lower than their usual total meat and poultry intake prior to the study (4.5 serves/day, SD = 2.0). As expected, meat and poultry intake was zero serves/day during the study for participants in the vegetarian group which was lower than their average intake of 5.3 (SD = 2.6) serves/day prior to the study.

**Table 4:** Food group and nutrient intake at baseline and week-10 according to intervention group.

		Total population		Flexitarian (*n* = 40)		Vegetarian (*n* = 38)		*p* values		
	Recommended intake^1^	Week 0	Week 10	Week 0	Week 10	Week 0	Week 10	Time	Diet	Time × diet
Food groups										
Fruit (serves/day)		1.6 ± 1.1	1.6 ± 1.0	1.6 ± 1.1	1.6 ± 0.9	1.7 ± 1.1	1.7 ± 1.1	0.924	0.488	0.627
Vegetables (serves/day)		2.2 ± 1.1	2.8 ± 1.3	2.2 ± 1.4	2.7 ± 1.5	2.1 ± 0.9	2.9 ± 1.1	<0.001	0.902	0.260
Cereals (serves/day)		2.0 ± 1.4	2.0 ± 1.1	2.0 ± 1.5	1.9 ± 1.0	2.0 ± 1.4	2.2 ± 1.2	0.616	0.676	0.552
Meat and Poultry (serves/week)		4.9 ± 2.8	1.7 ± 1.7	4.5 ± 2.9	3.3 ± 0.5	5.3 ± 2.6	0.0 ± 0.0	**<0.001**	**<0.001**	**<0.001**
Seafood (serves/week)		1.1 ± 1.2	0.0 ± 0.0	1.0 ± 1.2	0.0 ± 0.0	1.3 ± 1.3	0.0 ± 0.0	**<0.001**	0.183	0.183
Macronutrients										
Energy (kJ/day)		7,687 ± 2,289	7,079 ± 2,230	7,917 ± 2,262	7,032 ± 1978	7,456 ± 2,319	7,125 ± 2,482	**0.010**	0.644	0.276
Protein (g/day)		81.1 ± 25.8	62.8 ± 20.2	83.1 ± 27.6	63.4 ± 15.2	79.1 ± 24.1	62.2 ± 24.4	**<0.001**	0.513	0.677
Fat (g/day)		79.3 ± 26.5	71.7 ± 25.6	8.3 ± 27.9	71.9 ± 21.9	75.3 ± 24.7	71.6 ± 29.1	**0.006**	0.383	0.197
Saturated fat (g/day)		35.7 ± 13.7	35.0 ± 16.8	36.1 ± 13.4	35.6 ± 19.3	35.3 ± 14.1	34.4 ± 14.1	0.692	0.697	0.889
Monounsaturated fat (g/day)		27.6 ± 8.9	24.4 ± 8.8	28.7 ± 9.5	25.5 ± 8.6	26.5 ± 8.3	23.3 ± 9.0	**0.001**	0.209	0.998
Polyunsaturated fat (g/day)		23.9 ± 8.9	22.2 8.8	25.5 ± 8.9	22.2 ± 8.0	22.4 ± 8.6	22.2 ± 9.7	0.053	0.326	0.129
Carbohydrate (g/day)		187 ± 67.5	186 ± 67.3	194 ± 62.2	187 ± 68.8	181 ± 72.6	185 ± 66.7	0.814	0.582	0.469
Fibre (g/day)		26.0 ± 11.3	27.6 ± 9.8	28.5 ± 10.9	27.6 ± 8.8	23.5 ± 11.3	27.7 ± 10.7	0.123	0.237	**0.013**
Sucrose (g/day)		25.0 ± 14.1	24.3 ± 11.2	27.8 ± 12.6	25.7 ± 10.3	22.2 ± 15.1	22.9 ± 12.0	0.489	0.094	0.282
Fructose (g/day)		19.7 ± 9.4	19.2 ± 8.8	21.0 ± 7.7	20.1 ± 7.5	18.3 ± 10.8	18.3 ± 9.9	0.610	0.269	0.490
Vitamins										
Folate (μg/day)^2^	320	449 ± 238	459 ± 184	507 ± 250	450 ± 178	392 ± 212	468 ± 191	0.716	0.221	**0.003**
Niacin (mg/day)	11–12	20.1 ± 6.9	14.7 ± 4.9	20.3 ± 7.2	15.1 ± 4.6	19.9 ± 6.5	14.3 ± 5.2	**<0.001**	0.552	0.681
Riboflavin (mg/day)	0.9–1.1	1.8 ± 0.8	1.6 ± 0.7	1.9 ± 0.8	1.6 ± 0.6	1.8 ± 0.8	1.7 ± 0.8	**<0.001**	0.773	0.194
Thiamine (mg/day)	0.9–1.0	2.4 ± 2.6	2.0 ± 1.7	2.5 ± 2.6	1.8 ± 1.6	2.4 ± 2.6	2.1 ± 1.8	**0.023**	0.883	0.441
Vitamin B_6_ (mg/day)	1.1	1.9 ± 0.7	1.6 ± 0.6	2.0 ± 0.7	1.7 ± 0.5	1.8 ± 0.8	1.5 ± 0.7	**<0.001**	0.174	0.901
Vitamin B_12_ (μg/day)	2.0	3.2 ± 1.7	1.8 ± 1.2	3.0 ± 1.7	2.0 ± 0.8	3.4 ± 1.7	1.6 ± 1.4	**<0.001**	0.992	0.016
Vitamin C (mg/day)	30	86 ± 43	81 ± 41	87 ± 39	81 ± 33	84 ± 47	80 ± 49	0.250	0.846	0.737
Minerals										
Calcium (mg/day)	840	760 ± 428	717 ± 362	777 ± 419	670 ± 283	743 ± 433	764 ± 425	0.273	0.763	0.134
Iron (mg/day)	6–8	13.2 ± 5.3	12.1 ± 4.0	13.7 ± 5.3	12.0 ± 3.6	12.6 ± 5.3	12.2 ± 4.4	**0.039**	0.611	0.216
Sodium (mg/day)	<2,000^3^	2061 ± 727	1,682 ± 601	2096 ± 762	1,624 ± 508	2026 ± 697	1742 ± 683	**<0.001**	0.860	0.257
Zinc (mg/day)	6.5–12	10.8 ± 3.5	9.2 ± 2.9	11.3 ± 3.7	9.7 ± 2.4	10.2 ± 3.2	8.8 ± 3.3	**<0.001**	0.122	0.795

Data is presented as mean ± standard deviations, *p*-values are derived from linear mixed effect models. Significant findings (*p* < 0.05) are highlighted in bold text. Nutrient analysis for macronutrients, vitamins and minerals is derived from a self-administered food-frequency questionnaire, and serves of food groups is entered in a free text at the end of the food frequency questionnaire.

1 Recommended intakes refer to the estimated average requirements for micronutrients from the Australia/New Zealand Nutrient Reference Values, recommended intakes are not presented for macronutrients as these more appropriately used as an individual recommendation rather than applied to groups (46). In the case that requirements differ for males and females, this is presented as a range with females having the lower and males the upper recommended intake, except in the case of iron where females have higher recommended intakes.

2 Expressed as folate equivalents (taking into account differential bioavailability of the natural food-form of folate and folic acid in fortified foods).

3 The adequate intake for sodium is 460–920 mg/day, but a more appropriate target for health is defined as <2,000 mg/day in the nutrient reference values.

Changes to nutrient intake that were specific to the intervention arm included fibre, which increased in the vegetarian group only (time x diet interaction, *p* < 0.001; Tukey HSD post-hoc analysis, *p* = 0.029) while no significant change was found in the flexitarian group. Vitamin B_12_ increased significantly in the flexitarian group (time x diet interaction, *p* = 0.016; Tukey HSD post-hoc analysis, *p* = 0.001), but decreased in the vegetarian group.

Other changes in nutrient intake were seen in the total population including a decrease in protein, total fat, monounsaturated fat, niacin, riboflavin, thiamine, vitamin B_6_, iron, sodium, and zinc intake. It should however be noted that a decline in absolute nutrient intake is expected with the decline in energy intake that was seen in the total population (t_0_: 7687 kJ/day, SD = 2,289; t_10_: 7079 kJ/day, SD = 2,230). As highlighted in Table 4, group averages of nutrient intake also remained higher than recommended intakes except in the case of vitamin B_12_ in the vegetarian group.

Analysis of the week-22 survey (*n* = 64, 83%) found that vegetable intake remained marginally higher with average intakes of 2.5 serves per day at t_22_ compared to 2.2 serves per day at t_0_ in both intervention groups (*p* = 0.048). Weekly intake of meat and poultry remained lower than t_0_ values in the vegetarian group, with an average of 3.8 serves per week at t_22_ compared to 5.3 serves per week at t_0_ (*p* < 0.001; Supplementary Table 6). Nutrient intakes followed a similar pattern to the primary endpoint analysis, though intakes of niacin, riboflavin, thiamine, and calcium remained lower than baseline levels of intake.

### 3.6. Adverse events

One adverse event occurred during the dietary intervention, with gastrointestinal side effects (altered bowel habit, bloating, discomfort) related to the PBMA provided which required discontinuation from the study after confirmation and discussion with researchers. The trial protocol requires participants to complete the study in a household unit, which meant that the other participant in this household was also discontinued. No serious adverse events were recorded.

## 4. Discussion

Monitoring adherence is a crucial aspect of dietary interventions. Indeed, the relative success of trials in achieving desired endpoints depends on whether participants adhered to the prescribed intervention, yet this is often overlooked (11). We report comprehensive efforts to ensure that participants adhered to the dietary intervention, which was informed by conceptualizing adherence as a behaviour and utilizing a behaviour change framework (21). Strategies to encourage adherence included regular contact with participants through several communication avenues (email, text, Facebook), text message reminders, social support, cooking education, and the reward of food provision (28–30). This ensured that participants were supported to have the capability (e.g., cookbooks to enhance knowledge and skills), opportunity (e.g., social opportunity by competing the study with a household partner), and motivation (e.g., cessation of study food if they were consistently non-adherent) to regularly consume their red meat or PBMAs and maintain a basal vegetarian diet (31). These are tangible examples of behaviour change techniques, the active components that bring about change in behaviour (32). As a result, excellent adherence to the intervention was seen in the PREDITION trial, with a mean score of 91.5 (SD = 9.0) for the total population out a maximum possible score of 100. Responses in the exit survey reinforce the high adherence scores seen, with most participants finding it easy to adhere to the predominantly vegetarian basal diet (91% agreeing or strongly agreeing).

Of interest is that participants in the flexitarian group demonstrated greater adherence – that is both consuming all allocated red meat and abstaining from other red and white meat or seafood. Mean adherence scores were 96.1 (SD = 4.6) in the flexitarian group and 86.7 (SD = 10.0) in the vegetarian group, and we saw a marked decline in participants on the vegetarian group meeting adherence requirements from around week-7 onwards. These findings differ to those reported from the SWAP-MEAT study, one of the only other dietary interventions comparing a diet containing meat or PBMAs. Although a comparable adherence score was not used in the SWAP-MEAT study, authors reported similar serves of meat and PBMAs consumed across the intervention with presumably similar adherence between intervention groups (33). Participants were however required to consume greater portions of meat or PBMA and more frequently in the SWAP-MEAT study (at least 2 serves per day), which may partly explain greater adherence in the flexitarian compared to vegetarian groups of the current study where participants had only a low-to-moderate red meat intake reflective of a modern flexitarian diet (17).

Varying psychological, demographic, and external motivational factors have been found to influence adherence to study diets both here, and in previous research (12, 13). It has been proposed that motivation drives every failure or success in the dietary changes which are required in adhering to a prescribed intervention diet (34). While it could be argued that improved adherence on the flexitarian group in the PREDITION trial is expected given that participants were regularly consuming meat at the time of recruitment (and for at least 2 months prior), it should be noted that a leading motivation for participants joining this study was the opportunity to try a vegetarian diet (*n* = 27, 35% expressed that trying plant-based eating was a main interest in taking part in the study). Indeed, our findings align with previous research where younger adults are interested in reducing meat or attempting a meat free diet, but may struggle to adopt this pattern long term (16). There is a wealth of evidence which attempts to understand the “best” diet for human health, for example a comparison of vegetarian diets or those containing meat on cardiovascular risk factors (33), or the effects of ketogenic and Mediterranean diets on markers of glycemic control (35). It is increasingly clear that the composition of these different diets is less relevant than how well an individual can adhere to that diet (9, 10, 36). In this context, differences in adherence observed between the two intervention groups is important not only for later interpreting health outcomes of the PREDITION trial, but also for informing healthy, sustainable dietary patterns for young adults.

We considered whether participants’ subjective experience of the dietary intervention and food provided may in part explain these differences in adherence. Qualitative analyses revealed similar themes across intervention groups, with both positive and negative experiences. Participants reported that they enjoyed the quality of red meat, but some participants did not enjoy the quantity of red meat they had to consume which contrasts the mix of meat and poultry consumed in their habitual diet. We found mixed reviews regarding enjoyment of the PBMAs in the vegetarian group, both enjoying and disliking the taste of the products which was supported in quantitative analyses. Most participants in the flexitarian group felt satisfied with the red meat provided and enjoyed eating it (90%), whereas only 58% of the vegetarian group either agreed or strongly agreed with statements regarding enjoyment and satisfaction with the PBMAs which may in part explain differences in adherence observed between the intervention groups. Both intervention groups shared a want for more variety in the types of meat and PBMAs provided, however this was not possible as it was important to maintain equipoise between intervention groups (i.e., both treatment groups having an equal opportunity to succeed), by matching the cuts of red meat to PBMAs available on the market in New Zealand (primarily burgers and mince products). Quality was not explicitly measured in this review of participant’s satisfaction and enjoyment of the PBMAs or red meat, but is of course an important consideration in this context. We aimed to provide high quality PBMAs through selecting those nutritionally most similar to red meat, as there is currently a wide range of nutrient profiles in available PBMAs on the market (37, 38). We also took factors such as social norms (e.g., packaging, eating with others), texture and ease of preparation into consideration in selecting the highest quality meat alternatives available in the New Zealand market, as these are shown to influence an individual’s quality perceptions and acceptance of PBMAs (39). It is beyond the scope of the current investigation to comment on quality aspects of the range of PBMAs used in the PREDITION trial, though this is an important issue which is increasingly reported in the literature particularly with respect to nutrient profiles (37, 40, 41), but also other quality characteristics such as sensory profiles (42) and even impact on appetite or amino acid profiles (43).

Psychosocial factors related to eating behaviour were also measured by the Positive Eating Scale. This tool is designed to investigate the positive aspects of eating in non-clinical populations, and captures people’s experiences of eating, including the satisfaction and pleasure they experiencing during eating (14). Notably, participants’ positive eating experiences and the satisfaction they experience while eating increased during the dietary intervention. We did not observe differences in how the flexitarian and vegetarian groups responded over time, except for a trend towards higher pleasure subscale scores in the flexitarian group at week 10. To the best of our knowledge, this is the first time that the PES has been used in the context of a dietary intervention. Amongst the increasing concern and confusion around what people should be eating, it is encouraging to see a shift in positive experiences and satisfaction derived from eating during the PREDITION trial.

In attempts to increase consistency and comparability between intervention groups, one of the goals of the PREDITION trial was to encourage participants towards eating a healthy basal vegetarian diet. Common undesirable eating behaviours amongst young adults include low intakes of fruits, vegetables, and wholegrains, frequent consumption of discretionary foods (e.g., sugar-sweetened beverages and fast food), and irregular meal patterns (22, 23). These behaviours were targeted in the nutrition support package developed for the PREDITION trial (21). Participants received online nutrition education (meal planning resources, cooking videos, website links, educational videos) and recipe books (21), alongside weekly meal kit deliveries for three evening meals to encourage adoption of healthier dietary behaviours. We saw a shift in vegetable intake closer towards local dietary guidelines of 5–6 serves/day during the intervention, and this appeared to remain higher in participants who completed the week-22 survey, supporting the success of the nutrition support package not only during the intervention but also in achieving longer-lasting behaviour change (27). No change in fruit intake was seen, but this is not entirely surprising - although participants were encouraged to consume fruit more regularly, baseline intakes of 1.6 (SD = 1.1) serves/day were already relatively close to meeting the recommended two serves. Further, meal kits provided by the study included vegetables but not fruit, which reinforces the importance of food provision in changing behaviours during dietary intervention (44). Due to the nature of the FFQ used for this trial, we are limited in describing changes to other target nutrition behaviours during the intervention or total diet quality. A 3-day food record would have helped to overcome this limitation, though the FFQ was selected as a validated means to assess nutrients relevant to the primary outcome of the PREDITION trial which is a fatty acid analysis. Although participants were recording text entries into the Easy Diet Diary app twice weekly, the extent to which participants provided sufficient detail varied broadly and this is not appropriate for reporting dietary intake in the current analysis. While the findings do not synthesize new information around food group or nutrient intake with flexitarian or vegetarian dietary patterns more broadly, they do provide reassurance that participants changed their dietary behaviours and consumed a generally healthy basal vegetarian diet which met nutrient requirements.

There are strengths and limitations in the design and execution of this study. Despite difficult circumstances during the COVID-19 pandemic there was high participant retention and minimal missing data, with only 1 household unit withdrawing due to digestive discomfort and a 99% complete dataset for surveys during the 10-week intervention. This is likely in part attributed to the behaviour change framework used in the design and implementation of this research (21), which has been suggested to improve recruitment and retention in clinical trials (45). Having participants complete this study in pairs may have also contributed to the high completion and adherence rates seen in the study, as this is a layer of accountability for participants to remain engaged with the trial. At the same time, recruiting a pair of participants has the potential to inflate similarities within intervention arms and indeed we see some differences in baseline participant characteristics between the flexitarian and vegetarian groups (e.g., higher levels of education and better self-reported health in the flexitarian group) which could be related to recruiting pairs of participants. In an effort to mitigate the effect of the household pair on outcomes of interest, we have controlled for the fact that these data points are not independent by fitting mixed effects models with household pair nested within cohort as a random effect. Although some differences in baseline participant characteristics were observed despite random allocation, it should be noted that these were considered as explanatory variables with no effect found on behavioural outcomes like adherence or PES scores.

We have taken a “real-world” approach to the dietary intervention, not attempting to extrapolate the effects of red meat alone but rather the role of red meat in the context of a healthy, balanced diet. Studies in free-living individuals have the strength of greater generalizability, but do compromise adherence with less control over what participants actually consume. Our approach was to balance the two, which the high adherence scores support. We acknowledge that more meaningful conclusions regarding participant adherence and experience of the two diets could have been made with a cross-over trial design which should be considered in the future in the context of a modern flexitarian diet. Further, participants in this study were generally healthy and our findings may of course differ depending on population characteristics like age, socioeconomic status, and comorbidities. A meaningful comparison in dietary intervention studies requires both diets to have a fair opportunity to succeed (11), which we have attempted to achieve by providing a high quality and flexible basal diet, intervention foods with similar physical and functional formats, and matched nutrition support materials (e.g., recipe books adapted to the allocated red meat or PBMAs). It should be noted that despite these efforts, a barrier to achieving equivalence between the two groups are more advanced cooking skills required for some of the red meat cuts compared to the convenience of the PBMAs. Finally, these findings should not be generalized to flexitarian and vegetarian diets more broadly, with these terms used in this study as a broad overview of the dietary pattern our participants were following during the intervention. In particular, the term flexitarian should be considered with caution. There is no universally agreed definition for a flexitarian diet, and the inclusion of red meat is poorly defined. Using the term flexitarian for participants receiving the red meat allocation does align with some definitions considering meat reduction more generally (17), but contrasts others focusing more specifically on a reduction of red and processed meats (8). Similarly, participants regularly consuming PBMA in the vegetarian group of this study does not necessarily reflect a typical vegetarian diet, but can be summarized as a vegetarian diet.

## 5. Conclusion

We have presented a novel analysis of participant adherence, experiences, and related psychosocial factors during a randomised dietary intervention where participants received flexitarian (red meat) or vegetarian (PBMAs) allocations on top of a basal vegetarian diet. Participants demonstrated excellent adherence to the intervention overall, and positive eating experiences increased during the intervention. Of particular interest is that participants in the flexitarian group showed higher and more consistent adherence to the intervention. This finding has implications for the adoption of longer-term healthy diets for young adults beyond the current trial, and reinforces the need for more robust measures of adherence and related psychosocial factors to help inform the translation of findings from dietary intervention trials.

## Data availability statement

The raw data supporting the conclusions of this article will be made available by the authors, without undue reservation.

## Ethics statement

The studies involving human participants were reviewed and approved by New Zealand Ministry of Health and Disability Ethics Committee (20/STH/157). The patients/participants provided their written informed consent to participate in this study.

## Author contributions

AB, NG, SK, EB, TC, and DC-S designed the research. NG, AW, and LL conducted the research. RH and NG conducted the statistical analysis. NG drafted the manuscript. AB had primary responsibility for the final content of the manuscript. All authors contributed to the article and approved the submitted version.

## Funding

This research was funded by the New Zealand National Science Challenge (High Value Nutrition) and the New Zealand Ministry of Business, Innovation and Employment National [including funds from the Meat Industry Association Innovation Limited (a subsidiary of the New Zealand Meat Industry Association) and Beef and Lamb New Zealand Limited].

## Conflict of interest

The authors declare that the research was conducted in the absence of any commercial or financial relationships that could be construed as a potential conflict of interest.

## Publisher’s note

All claims expressed in this article are solely those of the authors and do not necessarily represent those of their affiliated organizations, or those of the publisher, the editors and the reviewers. Any product that may be evaluated in this article, or claim that may be made by its manufacturer, is not guaranteed or endorsed by the publisher.

## Supplementary material

The Supplementary material for this article can be found online at:

https://www.frontiersin.org/articles/10.3389/fnut.2023.1174726/full#supplementary-material
