# Supplementary material for: Adherence and eating experiences differ between participants following a flexitarian diet including red meat or a vegetarian diet including plant-based meat alternatives: findings from a 10-week randomised dietary intervention trial
Source: Front Nutr. 2023 Jun 14;10:1174726. doi: 10.3389/fnut.2023.1174726 (PMC10305861; doi:10.3389/fnut.2023.1174726)
Supplement: Supplementary file 1 [file Data_Sheet_1.docx]

Supplementary File 1 – Impacts of Covid-19 on the PREDITION trial

The PREDITION (pRotEin Diet SatisfacTION) Trial was conducted during the COVID-19 pandemic, which had varying degrees of impact on study participants depending on when they entered the trial as demonstrated in the schematic timeline below.

**Organization of participants in the PREDITION trial.**

Participants (n=80) were recruited as a “household unit”, which refers to a pair of individuals who cohabit. The 40 pairs were organized into 8 subgroups, each including 5 household units. Subgroups were formed chronologically, and were randomly allocated to receive either red meat (flexitarian group) or plant-based meat alternative (vegetarian group) inputs. Subgroups entered the 10-week intervention in a staggered series. Retrospectively, this has been defined as four ‘cohorots’, each comprising 2 sub-groups balanced for intervention allocation (Table 1). The further splitting of participants in subgroup 7 is clarified in the following paragraph.

**Table 1. Organization and Timeline of participants in the PREDITION trial**

| Cohort | Entry into intervention | Subgroup | Allocation |
| --- | --- | --- | --- |
| 1 | June 2021 | 1 (n=10) | Flexitarian |
|  |  | 2 (n=10) | Vegetarian |
| 2 | August 2021 | 3 (n=10) | Vegetarian |
|  |  | 4 (n=10) | Flexitarian |
| 3 | February 2022 | 5 (n=10) | Flexitarian |
|  |  | 6 (n=10) | Vegetarian |
| 4 | March 2022 | 7a (n=4) | Vegetarian |
|  |  | 7b (n=6) | Vegetarian |
|  |  | 8 (n=10) | Flexitarian |

**Differential impacts of Covid-19 on participants during the PREDITION trial.**

Participants in subgroup one were unaffected by Covid. A strict lockdown (alert level 4, “shelter in place”) was imposed by the Government in August 2021 when community transmission of the Delta variant was detected. This coincides with the final week of the dietary intervention for participants in subgroup 2. The 10-week follow up data collected in visit (anthropometry, blood pressure) could not be collected for subgroup 2, but blood collection and questionnaires were still completed. One participant in subgroup 2 was not able to complete the final blood collection as they had left Auckland at the onset of the strict level-4 lockdown, but they completed all online questionnaires. Participants in subgroups 3 and 4 entered the dietary intervention prior to the level-4 lockdown, but were required to stay at home for the majority of the 10-week intervention (alert levels 4 and 3).

The strict alert levels were replaced by a traffic light system (Covid Protection Framework) in 2022 when community transmission of the Omicron variant was detected. There was more flexibility to move and engage with normal activities during “red” and “orange” alert levels, but participants were having to self-isolate if they or someone they were living with tested positive for Covid-19. Individual experiences with Covid-19 were far broader for participants starting the trial in 2022 compared to participants completing the trial in 2021. There were disruptions in starting participants at the time that Omicron was detected in the community, as participants who had been randomised to an intervention arm and scheduled to start the trial were testing positive for Covid-19. These participants had to delay starting the trial to ensure they were still on the intervention arm they were allocated to, which meant that a subgroup 7 was split into a group of 4 (subgroup 7a) and 6 (subgroup 7b). If participants were required to isolate at the time of their final 10-week follow-up period, the dietary intervention was extended for an extra week with food provided.


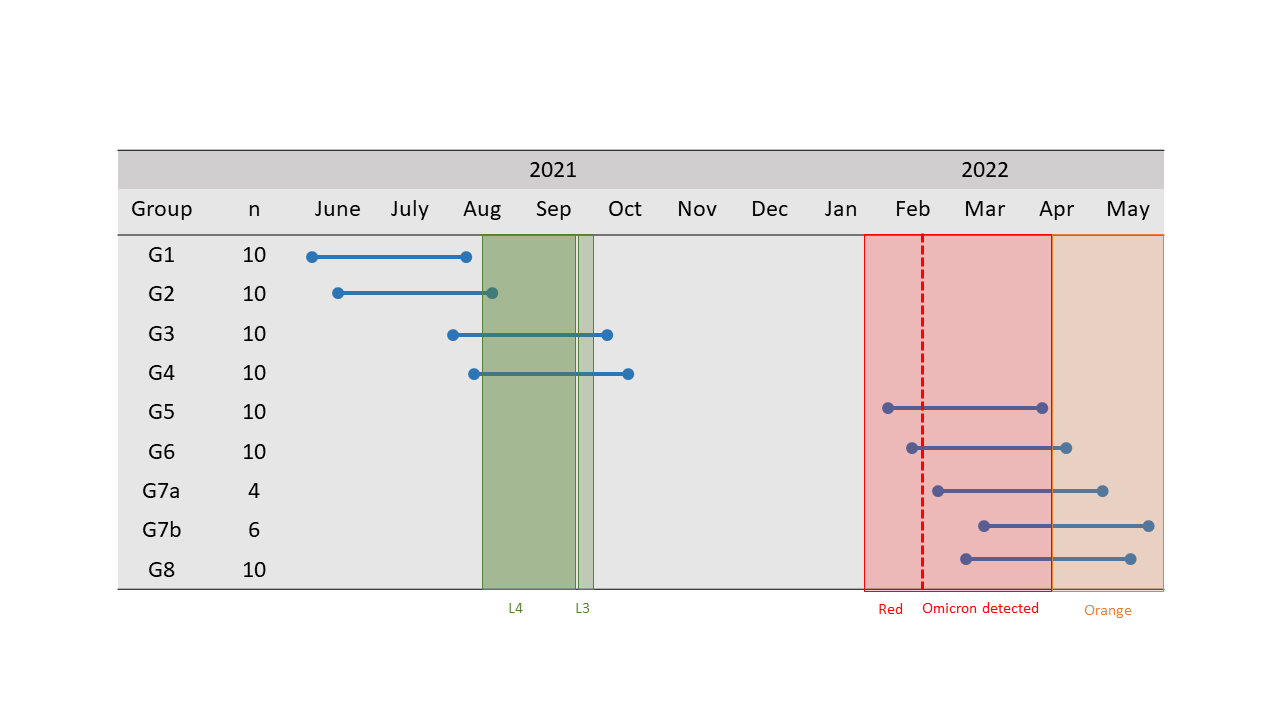


We collected data from participant self-reports, recording whether participants tested positive for Covid-19 or were required to self-isolate for Covid-19 during the 10-week dietary intervention. Retrospectively grouping participants into cohorts (2 subgroups per cohort, 1 randomly allocated to flexitarian and 1 randomly allocated to vegetarian) reflects similar experiences of Covid-19, and is used as a confounding variable in statistical analyses.
