# Supplementary material for: Adherence and eating experiences differ between participants following a flexitarian diet including red meat or a vegetarian diet including plant-based meat alternatives: findings from a 10-week randomised dietary intervention trial
Source: Front Nutr. 2023 Jun 14;10:1174726. doi: 10.3389/fnut.2023.1174726 (PMC10305861; doi:10.3389/fnut.2023.1174726)
Supplement: Supplementary file 2 [file Data_Sheet_2.docx]

Supplementary File 2 - Supplementary Tables and Figures

**Supplementary Table 1. Questionnaire completion rate across time**

|  | Total population  (n = 78) | Flexitarian (n = 40) | Vegetarian  (n = 38) |
| --- | --- | --- | --- |
| Study lead in^1^ | 78 | 40 | 38 |
| Week 0^2^ | 78 | 40 | 38 |
| Week 2 | 77 | 39 | 38 |
| Week 5 | 76 | 38 | 38 |
| Week 7 | 78 | 40 | 38 |
| Week 10 | 77 | 39 | 38 |
| Week 22 | 64 | 30 | 34 |

^1^2 weeks before allocation, ^2^At allocation.

**Supplementary Figure 1. Change in positive eating scale scores over time according to intervention group**


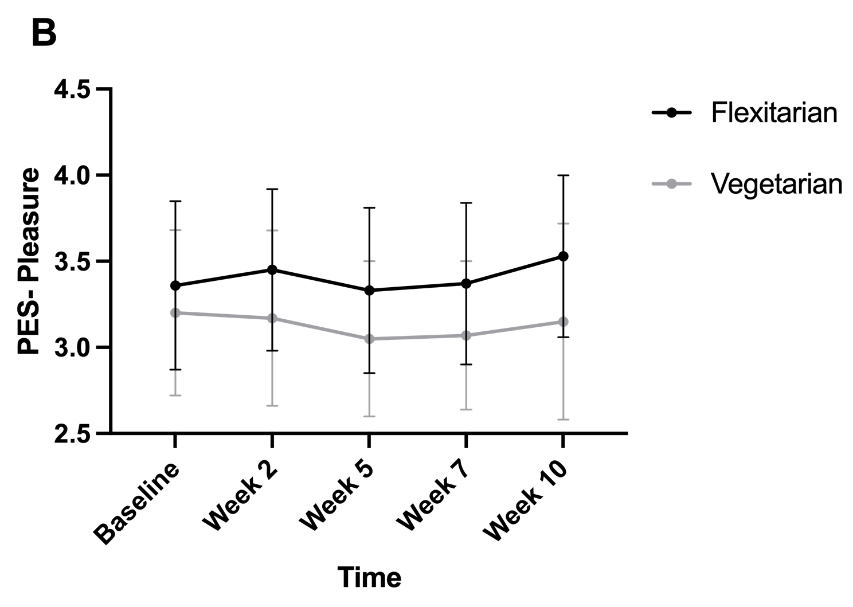

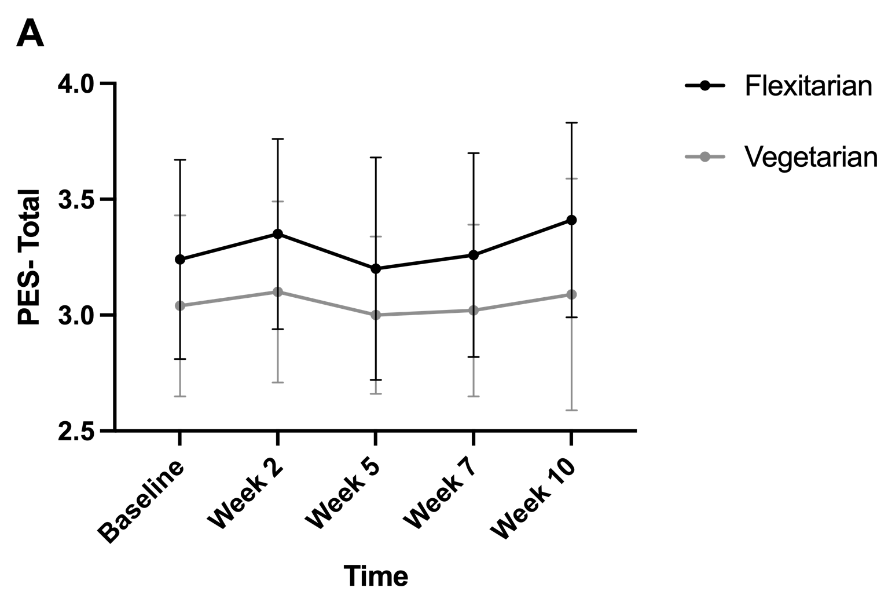

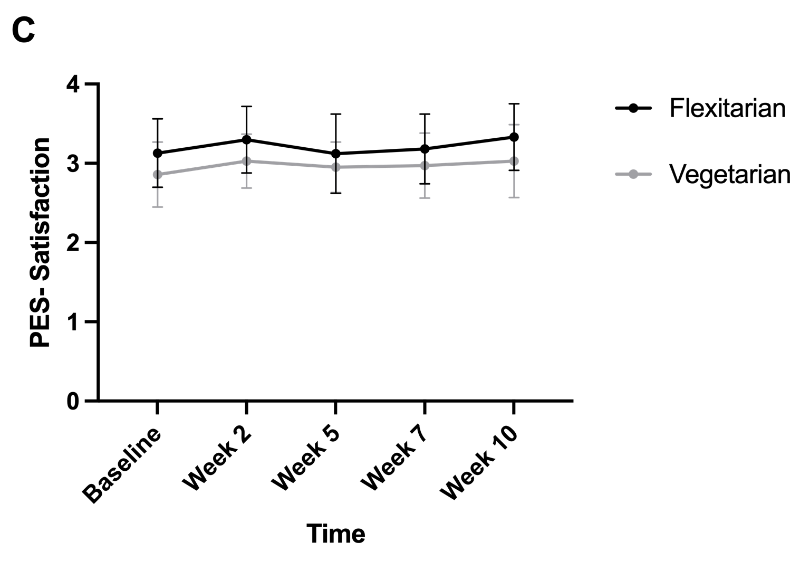


*


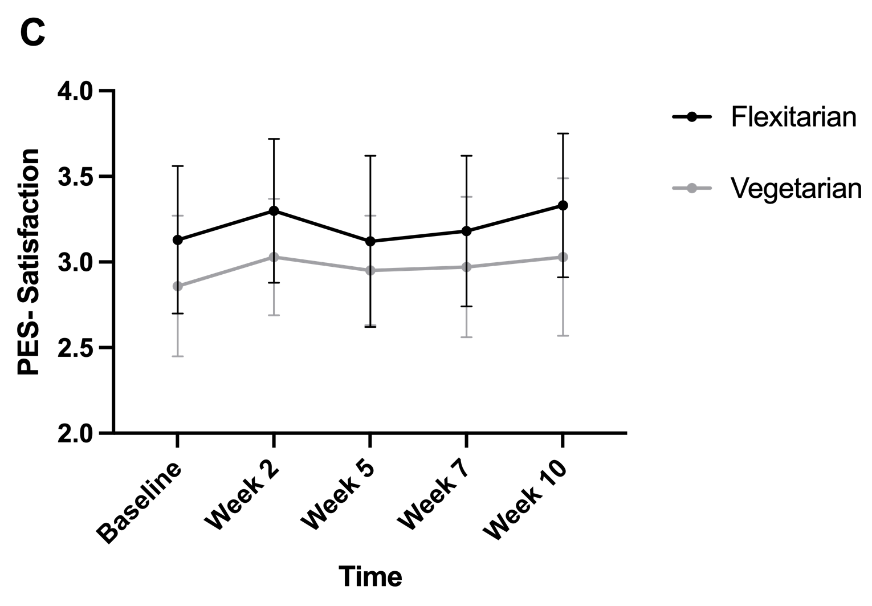


*

Data is presented as as mean and standard deviations. * Indicates a significant time difference (p <0.05) from baseline according to linear mixed models.

**Supplementary Table 2. Estimates derived from linear mixed effect models comparing differences in positive eating scores between intervention arms and across time.**

|  | **Positive Eating Scale – total score** | | | | **Positive Eating Scale – Pleasure subscale** | | | | **Positive Eating Scale – Satisfaction subscale** | | | |
| --- | --- | --- | --- | --- | --- | --- | --- | --- | --- | --- | --- | --- |
|  | Estimate (SE) | *t* | *df* | *P* value | Estimate (SE) | *t* | *df* | *P* value | Estimate (SE) | *t* | *df* | *P* value |
| **Vegetarian group** | -0.25 (0.11) | -2.35 | 65.16 | **0.022** | -0.19 (0.13) | -1.51 | 66.85 | 0.135 | -0.2 (0.11) | -1.77 | 72.12 | 0.081 |
| **Time** |  |  |  |  |  |  |  |  |  |  |  |  |
| Week 2 | 0.07 (0.06 | 1.08 | 329.91 | 0.280 | 0.06 (0.08) | 0.77 | 329.94 | 0.442 | 0.07 (0.07) | 1.05 | 331.87 | 0.294 |
| Week 5 | 0.02 (0.06) | 0.26 | 330.01 | 0.792 | 0.02 (0.08) | 0.28 | 330.04 | 0.783 | 0.02 (0.07) | 0.36 | 331.96 | 0.721 |
| Week 7 | 0.03 (0.06) | 0.50 | 329.76 | 0.616 | 0.02 (0.08) | 0.25 | 329.78 | 0.801 | 0.03 (0.07) | 0.48 | 331.72 | 0.632 |
| Week 10 | 0.15 (0.06) | 2.34 | 329.85 | **0.020** | 0.14 (0.08) | 1.77 | 329.87 | 0.077 | 0.16 (0.07) | 2.31 | 331.81 | **0.021** |
| **Time x Vegetarian group** |  |  |  |  |  |  |  |  |  |  |  |  |
| Week 2 | 0.00 (0.09) | -0.01 | 329.84 | 0.995 | -0.09 (0.11) | -0.85 | 329.86 | 0.398 | 0.00 (0.10) | -0.02 | 331.79 | 0.986 |
| Week 5 | -0.05 (0.09) | -0.51 | 329.89 | 0.611 | -0.17 (0.11) | -1.58 | 329.91 | 0.114 | -0.05 (0.10) | -0.56 | 331.84 | 0.575 |
| Week 7 | -0.05 (0.09) | -0.53 | 329.76 | 0.594 | -0.16 (0.11) | -1.45 | 329.78 | 0.147 | -0.05 (0.10) | -0.51 | 331.72 | 0.611 |
| Week 10 | -0.09 (0.09) | -1.04 | 329.80 | 0.300 | -0.19 (0.11) | -1.74 | 329.82 | 0.084 | -0.10 (0.10) | -1.04 | 331.76 | 0.297 |
| **Exercise** |  |  |  |  |  |  |  |  | - | - | - | - |
| Sedentary | 0.53 (0.13) | 4.1 | 364 | **<0.001** | 0.63 (0.15) | 4.08 | 364 | **<0.001** |  |  |  |  |
| Moderate | 0.48 (0.08) | 6.0 | 362.5 | **<0.001** | 0.61 (0.09) | 6.5 | 362 | **<0.001** |  |  |  |  |
| Vigorous | 0.40 (0.09) | 4.28 | 351.6 | **<0.001** | 0.54 (0.11) | 4.9 | 348 | **<0.001** |  |  |  |  |
| **Three-Factor Eating Questionnaire score** | 0.76 (0.23) | 3.33 | 364.1 | **0.001** | 1.17 (0.27) | 4.33 | 364 | **<0.001** | 0.64 (0.24) | 2.65 | 367.85 | **0.008** |
| **Self-efficacy** | 0.04 (0.01) | 4.28 | 297 | **<0.001** | 0.04 (0.01) | 4.28 | 297 | **<0.001** | - | - | - | - |
| **Self-reported health** | - | - | - | - | - | - | - | - |  |  |  |  |
| Very good |  |  |  |  |  |  |  |  | 0.08 (0.06) | 1.43 | 351.6 | 0.152 |
| Excellent |  |  |  |  |  |  |  |  | 0.25 (0.07) | 3.58 | 348.17 | **<0.001** |

Variables reported here were included in the final models, unless marked with “-”. Please see Supplementary Table 6 for details regarding the model selection process using AICc. Comparator variables for categorical variables include red meat (intervention), not isolation (Covid-19 isolation) baseline (time), no exercise (exercise), fair/poor health (self-reported health). All analyses controlled for household clustering.

**Supplementary Table 3. AICc table demonstrating model selection process for adherence scores**

| (Intercept) | Age | BMI | Intervention | Education | Exercise | Health | Isolating | Relationship | SE | Sex | TFEQ | AICc |
| --- | --- | --- | --- | --- | --- | --- | --- | --- | --- | --- | --- | --- |
| **Adherence** |  |  |  |  |  |  |  |  |  |  |  |  |
| 3.00 |  |  | + |  |  |  | + |  |  |  | 1.63 | 237.33 |
| 2.25 |  |  | + |  |  |  |  | + |  |  | 1.89 | 237.55 |
| 2.63 |  |  | + |  |  |  |  |  |  |  | 1.85 | 237.97 |
| 2.63 |  |  | + |  |  |  | + | + |  |  | 1.69 | 238.28 |
| 4.81 |  | -0.086 | + |  |  |  | + |  |  |  | 2.17 | 238.70 |
| 3.85 |  |  | + |  |  |  | + |  |  |  |  | 238.89 |
| 3.21 |  |  | + |  |  |  |  | + |  |  |  | 239.77 |
| 2.99 |  |  | + |  |  |  | + |  |  | + | 1.84 | 239.87 |
| 4.42 |  | -0.085 | + |  |  |  | + | + |  |  | 2.22 | 239.90 |
| 3.52 |  |  | + |  |  |  | + | + |  |  |  | 239.91 |

Abbreviations: BMI, body mass index; SE, self efficacy, TFEQ; three factor eating questionnaire. Health refers to self-reported health, Relationship refers to whether household pairs are in a relationship or flatting.

**Supplementary Table 4. AICc table demonstrating model selection process for Positive Eating Scale scores**

| (Intercept) | Age | BMI | Intervention | Education | Exercise | Health | Isolating | Relationship | SE | Sex | TFEQ | AICc |
| --- | --- | --- | --- | --- | --- | --- | --- | --- | --- | --- | --- | --- |
| **Total scores** |  |  |  |  |  |  |  |  |  |  |  |  |
| 1.56 |  |  | + |  | + |  |  |  | 0.03 |  | 0.76 | 250.67 |
| 2.64 |  |  | + |  | + |  |  |  |  |  | 0.52 | 253.23 |
| 1.43 |  |  | + |  | + |  |  |  | 0.03 | + | 0.73 | 253.91 |
| 1.51 |  |  | + |  | + |  | + |  | 0.03 |  | 0.78 | 254.15 |
| 1.48 |  |  |  |  | + |  |  |  | 0.02 |  | 0.75 | 254.76 |
| 1.53 |  |  | + |  | + | + |  |  | 0.02 |  | 0.93 | 254.81 |
| 2.48 |  |  | + |  | + | + |  |  |  |  | 0.74 | 255.01 |
| 1.60 |  |  | + |  | + |  |  | + | 0.03 |  | 0.76 | 255.09 |
| 2.51 |  |  |  |  | + |  |  |  |  |  | 0.52 | 255.29 |
| 2.93 |  |  | + |  | + |  |  |  |  |  |  | 255.32 |
| **Satisfaction scale** |  |  |  |  |  |  |  |  |  |  |  |  |
| 2.65 |  |  |  |  |  | + |  |  |  |  | 0.57 | 294.64 |
| 2.77 |  |  | + |  |  | + |  |  |  |  | 0.55 | 294.96 |
| 2.39 |  |  |  |  | + | + |  |  |  |  | 0.64 | 295.21 |
| 2.51 |  |  | + |  | + | + |  |  |  |  | 0.62 | 295.66 |
| 2.97 |  |  |  |  |  | + |  |  |  |  |  | 296.71 |
| 3.08 |  |  | + |  |  | + |  |  |  |  |  | 296.81 |
| 2.37 |  |  |  |  | + | + |  |  |  | + | 0.57 | 297.39 |
| 2.49 |  |  | + |  | + | + |  |  |  | + | 0.55 | 297.51 |
| 2.83 |  | -0.02 |  |  | + | + |  |  |  | + | 0.67 | 297.59 |
| 2.80 |  | -0.02 |  |  | + | + |  |  |  |  | 0.75 | 297.76 |
| **Pleasure scale** |  |  |  |  |  |  |  |  |  |  |  |  |
| 0.95 |  |  | + |  | + |  |  |  | 0.04 |  | 1.17 | 377.84 |
| 0.88 |  |  | + |  | + |  | + |  | 0.04 |  | 1.19 | 378.88 |
| 0.85 |  |  |  |  | + |  |  |  | 0.03 |  | 1.16 | 381.02 |
| 0.99 |  |  | + |  | + |  |  | + | 0.04 |  | 1.17 | 382.03 |
| 0.78 |  |  |  |  | + |  | + |  | 0.03 |  | 1.19 | 382.55 |
| 0.87 |  |  | + |  | + |  |  |  | 0.04 | + | 1.15 | 383.45 |
| 0.87 |  |  | + |  | + |  | + | + | 0.04 |  | 1.20 | 383.52 |
| 0.93 |  |  | + |  | + | + | + |  | 0.03 |  | 1.18 | 383.71 |
| 1.02 |  |  | + |  | + | + |  |  | 0.04 |  | 1.13 | 384.23 |
| 0.80 |  |  | + |  | + |  | + |  | 0.04 | + | 1.17 | 384.42 |

Abbreviations: BMI, body mass index; SE, self efficacy, TFEQ; three factor eating questionnaire. Health refers to self-reported health, Relationship refers to whether household pairs are in a relationship or flatting.

**Supplementary Table 5. Exploratory analyses including week-22 survey data (including only those participants finishing the week-22 survey, n=65) - Estimates derived from linear mixed effect models comparing differences in positive eating scores between intervention arms and across time.**

|  | **Positive Eating Scale – total score** | | | | **Positive Eating Scale – Pleasure subscale** | | | | **Positive Eating Scale – Satisfaction subscale** | | | |
| --- | --- | --- | --- | --- | --- | --- | --- | --- | --- | --- | --- | --- |
|  | Estimate (SE) | *t* | *df* | *P* value | Estimate (SE) | *t* | *df* | *P* value | Estimate (SE) | *t* | *df* | *P* value |
| **Vegetarian group** | -0.25 | -2.33 | 68.96 | **0.023** | -0.25 | -2.17 | 81.72 | **0.033** | -0.19 | -1.50 | 68.42 | 0.139 |
| **Time** |  |  |  |  |  |  |  |  |  |  |  |  |
| Week 2 | 0.07 | 1.05 | 390.9 | 0.293 | 0.08 | 1.12 | 392.7 | 0.262 | 0.06 | 0.76 | 390.8 | 0.447 |
| Week 5 | 0.02 | 0.27 | 391.0 | 0.785 | 0.02 | 0.23 | 392.8 | 0.817 | 0.02 | 0.29 | 390.9 | 0.774 |
| Week 7 | 0.03 | 0.19 | 390.8 | 0.625 | 0.04 | 0.62 | 392.5 | 0.537 | 0.02 | 0.25 | 390.7 | 0.801 |
| Week 10 | 0.15 | 2.25 | 390.8 | **0.025** | 0.17 | 2.27 | 392.6 | **0.024** | 0.13 | 1.74 | 390.8 | 0.083 |
| Week 22 | 0.03 | 0.18 | 391.8 | 0.632 | 0.04 | 0.56 | 393.7 | 0.782 | 0.03 | 0.36 | 391.7 | 0.720 |
| **Time x Vegetarian group** |  |  |  |  |  |  |  |  |  |  |  |  |
| Week 2 | 0.00 | 0.00 | 390.8 | 0.996 | 0.09 | 0.86 | 392.6 | 0.392 | -0.09 | -0.84 | 390.7 | 0.402 |
| Week 5 | -0.05 | -0.51 | 390.9 | 0.612 | 0.08 | 0.72 | 392.6 | 0.471 | -0.17 | -1.58 | 390.8 | 0.114 |
| Week 7 | -0.05 | -0.52 | 390.7 | 0.604 | 0.06 | 0.58 | 392.5 | 0.560 | -0.16 | -1.45 | 390.7 | 0.149 |
| Week 10 | -0.09 | -0.99 | 380.8 | 0.324 | 0.00 | -0.01 | 392.6 | 0.989 | -0.19 | -1.71 | 390.7 | 0.088 |
| Week 22 | -0.07 | -0.70 | 391.4 | 0.485 | -0.02 | -0.16 | 393.3 | 0.875 | -0.11 | -0.99 | 391.3 | 0.323 |
| **Exercise** |  |  |  |  | - | - | - | - |  |  |  |  |
| Sedentary | 0.45 | 3.59 | 424.7 | **<0.001** |  |  |  |  | 0.53 | 3.64 | 425.0 | **<0.001** |
| Moderate | 0.44 | 5.74 | 423.4 | **<0.001** |  |  |  |  | 0.58 | 6.42 | 423.3 | **<0.001** |
| Vigorous | 0.35 | 3.86 | 408.0 | **<0.001** |  |  |  |  | 0.48 | 4.51 | 408.6 | **<0.001** |
| **Three-Factor Eating Questionnaire score** | 0.72 | 3.29 | 423.7 | **0.001** | 0.45 | 1.86 | 428.0 | 0.063 | 1.19 | 4.67 | 424.1 | **<0.001** |
| **Self-efficacy** | 0.03 | 3.82 | 347.9 | **<0.001** | - | - | - | - | 0.04 | 4.48 | 349.8 | **<0.001** |
| **Self-reported health** | - | - | - | - |  |  |  |  | - | - | - | - |
| Very good |  |  |  |  | 0.19 | 3.44 | 390.3 | **0.001** |  |  |  |  |
| Excellent |  |  |  |  | 0.37 | 5.14 | 384.9 | **<0.001** |  |  |  |  |

Variables reported here were included in the final models, unless marked with “-”. Please see Supplementary Table 6 for details regarding the model selection process using AICc. Comparator variables for categorical variables include red meat (intervention), not isolation (Covid-19 isolation) baseline (time), no exercise (exercise), fair/poor health (self-reported health). All analyses controlled for household clustering.

**Supplementary Table 6.** **Exploratory analyses including week-22 survey data (including only those participants finishing the week-22 survey, n=65) – Changes to dietary intake from baseline to the 10-week and 22-week follow-up according to intervention group**

|  | Total population | | | Flexitarian (n=31) | | | Vegetarian (n=34) | | | P value | | |
| --- | --- | --- | --- | --- | --- | --- | --- | --- | --- | --- | --- | --- |
|  | Week 0 | Week 10 | Week 22 | Week 0 | Week 10 | Week 22 | Week 0 | Week 10 | Week 22 | Time | Diet | Time x Diet |
| **Food groups** |  |  |  |  |  |  |  |  |  |  |  |  |
| Fruit | 1.6 ± 1.1 | 1.6 ± 1.0 | 1.7 ± 1.0 | 1.6 ± 1.1 | 1.6 ± 0.9 | 1.8 ± 1.0 | 1.7 ± 1.1 | 1.7 ± 1.1 | 1.6 ± 1.0 | 0.926 | 0.845 | 0.391 |
| Vegetables | 2.2 ± 1.1 | 2.8 ± 1.3^a^ | 2.5 ± 1.2^a^ | 2.2 ± 1.4 | 2.7 ± 1.5 | 2.5 ± 1.4 | 2.1 ± 0.9 | 2.9 ± 1.1 | 2.5 ± 0.9 | **<0.001** | 0.983 | 0.878 |
| Cereals | 2.0 ± 1.4 | 2.0 ± 1.1 | 2.1 ± 1.1 | 2.0 ± 1.5 | 1.9 ± 1.0 | 2.3 ± 1.2 | 2.0 ± 1.4 | 2.2 ± 1.2 | 1.9 ± 1.0 | 0.660 | 0.399 | 0.445 |
| Meat and Poultry | 4.9 ± 2.8 | 1.7 ± 1.7 | 4.0 ± 3.1 | 4.5 ± 2.9 | 3.3 ± 0.5^b^ | 4.2 ± 3.1 | 5.3 ± 2.6 | 0.0 ± 0.0^b^ | 3.8 ± 3.2^b^,^e^ | <0.001 | 0.018 | **<0.001** |
| Seafood | 1.1 ± 1.2 | 0.0 ± 0.0^b^ | 1.0 ± 1.2 | 1.0 ± 1.2 | 0.0 ± 0.0 | 0.8 ± 1.2 | 1.3 ± 1.3 | 0.0 ± 0.0 | 1.2 ± 1.2 | **<0.001** | 0.271 | 0.437 |
| **Macronutrients** |  |  |  |  |  |  |  |  |  |  |  |  |
| Energy | 7687 ± 2289 | 7079 ± 2230 | 7568 ± 3686 | 7917 ± 2262 | 7032 ± 1978 | 7301 ± 2544 | 7456 ± 2319 | 7125 ± 2482 | 7812 ± 2826 | 0.142 | 0.949 | 0.148 |
| Protein | 81.1 ± 25.8 | 62.8 ± 20.2^b^ | 76.4 ± 25.4 | 83.1 ± 27.6 | 63.4 ± 15.2 | 74.0 ± 24.2 | 79.1 ± 24.1 | 62.2 ± 24.4 | 78.5 ± 26.6 | **<0.001** | 0.779 | 0.158 |
| Fat | 79.3 ± 26.5 | 71.7 ± 25.6 | 76.7 ± 25.9 | 8.3 ± 27.9 | 71.9 ± 21.9 | 75.7 ± 26.2 | 75.3 ± 24.7 | 71.6 ± 29.1 | 77.7 ± 25.9 | 0.056 | 0.526 | 0.148 |
| Saturated fat | 35.7 ± 13.7 | 25.0 ± 16.8 | 33.9 ± 14.7 | 36.1 ± 13.4 | 35.6 ± 19.3 | 33.3 ± 15.4 | 35.3 ± 14.1 | 34.4 ± 14.1 | 34.5 ± 14.3 | 0.402 | 0.962 | 0.746 |
| MUFA | 27.6 ± 8.9 | 24.4 ± 8.8^b^ | 26.9 ± 8.9 | 28.7 ± 9.5 | 25.5 ± 8.6 | 26.8 ± 8.9 | 26.5 ± 8.3 | 23.3 ± 9.0 | 27.1 ± 9.0 | **0.029** | 0.350 | 0.249 |
| PUFA | 23.9 ± 8.9 | 22.2 8.8 | 22.4 ± 8.6 | 25.5 ± 8.9 | 22.2 ± 8.0 | 22.1 ± 8.9 | 22.4 ± 8.6 | 22.2 ± 9.7 | 22.8 ± 8.4 | 0.067 | 0.550 | 0.076 |
| Carbohydrate | 187 ± 67.5 | 186 ± 67.3 | 188 ± 82.2 | 194 ± 62.2 | 187 ± 68.8 | 181 ± 72.0 | 181 ± 72.6 | 185 ± 66.7 | 194 ± 91.4 | 0.784 | 0.831 | 0.156 |
| Fibre | 26.0 ± 11.3 | 27.6 ± 9.8 | 26.7 ± 13.0 | 28.5 ± 10.9 | 27.6 ± 8.8 | 27.0 ± 13.5 | 23.5 ± 11.3 | 27.7 ± 10.7 | 26.3 ± 12.7 | 0.132 | 0.313 | 0.145 |
| Sucrose | 25.0 ± 14.1 | 24.3 ± 11.2 | 22.9 ± 11.2 | 27.8 ± 12.6 | 25.7 ± 10.3 | 24.2 ± 11.2 | 22.2 ± 15.1 | 22.9 ± 12.0 | 21.7 ± 11.2 | 0.059 | 0.147 | 0.551 |
| Fructose | 19.7 ± 9.4 | 19.2 ± 8.8 | 17.5 ± 7.7^c^ | 21.0 ± 7.7 | 20.1 ± 7.5 | 18.0 ± 7.5 | 18.3 ± 10.8 | 18.3 ± 9.9 | 17.0 ± 7.9 | **0.015** | 0.316 | 0.679 |
| **Vitamins** |  |  |  |  |  |  |  |  |  |  |  |  |
| Folate | 449 ± 238 | 459 ± 184 | 461 ± 256 | 507 ± 250 | 450 ± 178 | 475 ± 290 | 392 ± 212 | 468 ± 191 | 447 ± 224 | 0.817 | 0.241 | **0.022^d^** |
| Niacin | 20.1 ± 6.9 | 14.7 ± 4.9^b^ | 18.7 ± 6.8^b,e^ | 20.3 ± 7.2 | 15.1 ± 4.6 | 18.2 ± 6.1 | 19.9 ± 6.5 | 14.3 ± 5.2 | 19.2 ± 7.4 | **<0.001** | 0.841 | 0.288 |
| Riboflavin | 1.8 ± 0.8 | 1.6 ± 0.7^b^ | 1.6 ± 0.7^b^ | 1.9 ± 0.8 | 1.6 ± 0.6 | 1.5 ± 0.6 | 1.8 ± 0.8 | 1.7 ± 0.8 | 1.7 ± 0.8 | **0.001** | 0.975 | 0.085 |
| Thiamine | 2.4 ± 2.6 | 2.0 ± 1.7^b^ | 1.8 ± 1.7^b^ | 2.5 ±2.6 | 1.8 ± 1.6 | 1.5 ± 0.9 | 2.4 ± 2.6 | 2.1 ± 1.8 | 2.1 ± 2.2 | **0.003** | 0.369 | 0.390 |
| Vitamin B6 | 1.9 ± 0.7 | 1.6 ± 0.6^b^ | 1.8 ± 0.7 | 2.0 ± 0.7 | 1.7 ± 0.5 | 1.8 ± 0.7 | 1.8 ± 0.8 | 1.5 ± 0.7 | 1.8 ± 0.7 | **0.001** | 0.299 | 0.257 |
| Vitamin B12 | 3.2 ± 1.7 | 1.8 ± 1.2 | 2.9 ± 1.7 | 3.0 ± 1.7 | 2.0 ± 0.8^b^ | 2.7 ± 1.4 | 3.4 ± 1.7 | 1.6 ± 1.4^b^ | 3.0 ± 1.9^e^ | <0.001 | 0.839 | **0.033** |
| Vitamin C | 857 ± 429 | 805 ± 414 | 839 ± 511 | 873 ± 386 | 807 ± 334 | 840 ± 559 | 842 ± 473 | 803 ± 485 | 838 ± 471 | 0.707 | 0.992 | 0.961 |
| **Minerals** |  |  |  |  |  |  |  |  |  |  |  |  |
| Calcium | 760 ± 428 | 717 ± 362 | 667 ± 325^b^ | 777 ± 419 | 670 ± 283 | 601 ± 246 | 743 ± 433 | 764 ± 425 | 727 ± 377 | **0.007** | 0.414 | 0.163 |
| Iron | 13.2 ± 5.3 | 12.1 ± 4.0 | 12.4 ± 5.3 | 13.7 ± 5.3 | 12.0 ± 3.6 | 12.0 ± 5.3 | 12.6 ± 5.3 | 12.2 ± 4.4 | 12.7 ± 5.4 | 0.096 | 0.739 | 0.208 |
| Sodium | 2061 ± 727 | 1682 ± 601 | 2056 ± 859 | 2096 ± 762 | 1624 ± 508^b^ | 1908 ± 756^e^ | 2026 ± 697 | 1742 ± 683^b^ | 2190 ± 933 | <0.001 | 0.651 | **0.042** |
| Zinc | 10.8 ± 3.5 | 9.2 ± 2.9^b^ | 10.3 ± 3.4 | 11.3 ± 3.7 | 9.7 ± 2.4 | 10.2 ± 3.3 | 10.2 ± 3.2 | 8.8 ± 3.3 | 10.4 ± 3.5 | 0.001 | 0.249 | 0.056 |

^a^Higher than baseline according to Tukey post-hoc comparison (*p*<0.05), ^b^Lower than baseline according to Tukey post-hoc comparison (*p*<0.05), ^c^Lower than week-10 according to Tukey post-hoc comparison (*p*<0.05), ^d^Time x diet interaction was not significant according to Tukey post-hoc comparison (*p*>0.05), ^e^Higher than week-10 according to Tukey post-hoc comparison (*p*<0.05)

**Supplementary Figure 2. Model diagnostics from mixed effects models demonstrate that the normality assumption related to the conditional distribution of errors is satisfied.**

**
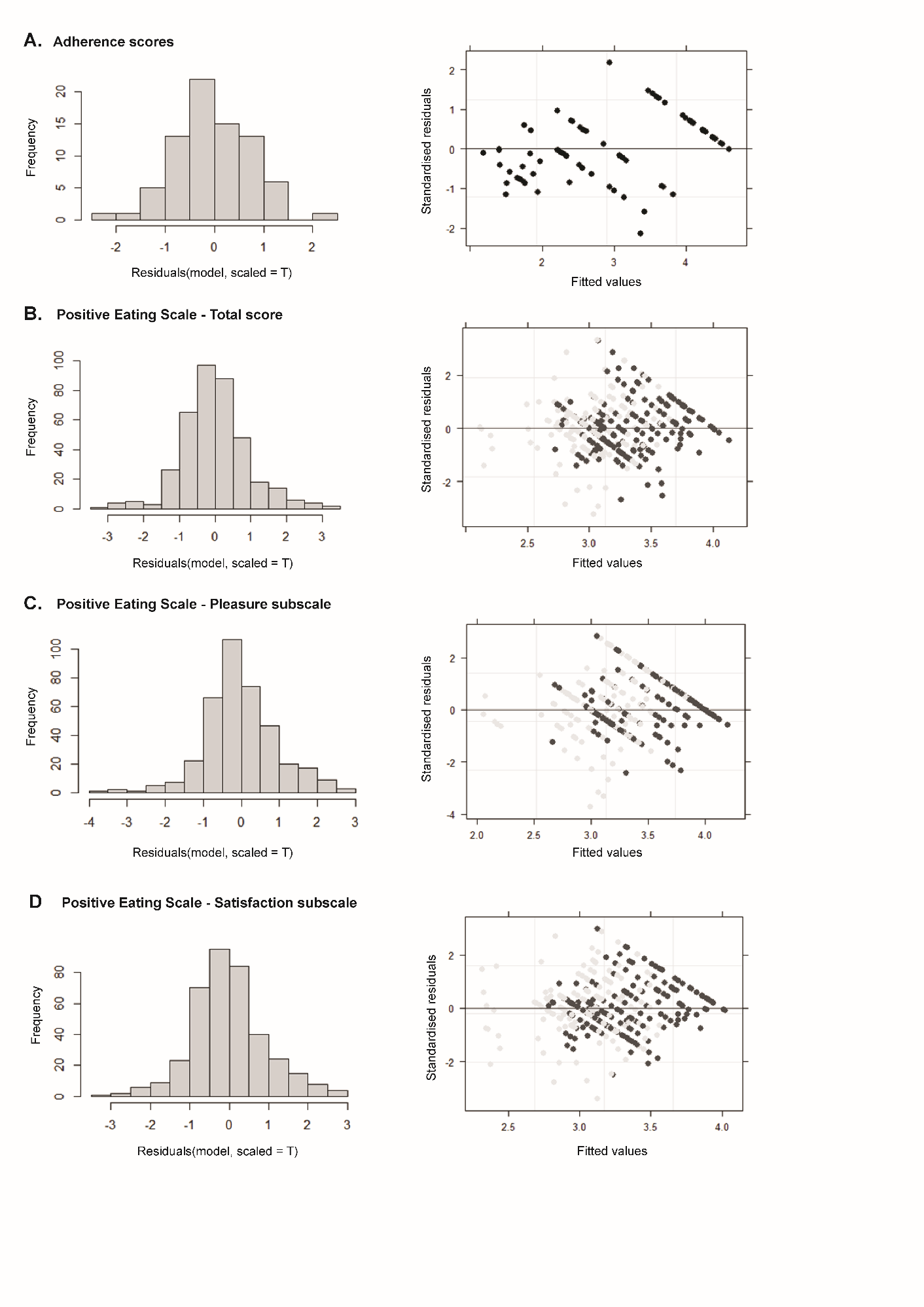
**
